# Genome size and ploidy influence angiosperm species' biomass under nitrogen and phosphorus limitation

**DOI:** 10.1111/nph.13881

**Published:** 2016-02-15

**Authors:** Maïté S. Guignard, Richard A. Nichols, Robert J. Knell, Andy Macdonald, Catalina‐Andreea Romila, Mark Trimmer, Ilia J. Leitch, Andrew R. Leitch

**Affiliations:** ^1^ School of Biological and Chemical SciencesQueen Mary University of LondonMile End RoadLondonE1 4NSUK; ^2^ Jodrell LaboratoryRoyal Botanic Gardens, KewRichmondSurreyTW9 3DSUK; ^3^ Department of Sustainable Soils and Grassland SystemsRothamsted ResearchHarpendenHertfordshireAL5 2JQUK

**Keywords:** C‐S‐R strategy, genome size, nitrogen, nutrient limitation, Park Grass, phosphate, plant community, polyploidy

## Abstract

- Angiosperm genome sizes (GS) range *c*. 2400‐fold, and as nucleic acids are amongst the most phosphorus‐ (P) and nitrogen (N)‐demanding cellular biomolecules, we test the hypothesis that a key influence on plant biomass and species composition is the interaction between N and P availability and plant GS.
- We analysed the impact of different nutrient regimes on above‐ground biomass of angiosperm species with different GS, ploidy level and Grime's C‐S‐R (competitive, stress‐tolerant, ruderal) plant strategies growing at the Park Grass Experiment (Rothamsted, UK), established in 1856.
- The biomass‐weighted mean GS of species growing on plots with the addition of both N and P fertilizer were significantly higher than that of plants growing on control plots and plots with either N or P. The plants on these N + P plots are dominated by polyploids with large GS and a competitive plant strategy.
- The results are consistent with our hypothesis that large genomes are costly to build and maintain under N and P limitation. Hence GS and ploidy are significant traits affecting biomass growth under different nutrient regimes, influencing plant community composition and ecosystem dynamics. We propose that GS is a critical factor needed in models that bridge the knowledge gap between biodiversity and ecosystem functioning.

## Introduction

The huge diversity of genome sizes (GS) encountered in plants has the potential to influence a plant's nutrient demands and hence its ability to grow in different environments varying in nutrient availability. Angiosperm GS (often referred to as 1C‐values = the amount of DNA in the unreplicated gametic nucleus) range an astonishing *c*. 2400‐fold, from 1C = 0.06 pg in *Genlisia tuberosa* (Fleischmann *et al*., 2014) to 1C = 152.2 pg in *Paris japonica* (Pellicer *et al*., 2010) (1 pg = 978 Mbp; the distribution of GS across > 7000 angiosperm species is shown in Supporting Information Fig. S1a). Angiosperm genomic diversity is also marked by the prevalence of polyploidy in the ancestry of most, perhaps all lineages. In addition, 15% of speciation events are estimated to involve polyploidy, 24% of species are recognisably polyploid from chromosome counts, and many species have polyploid cytotypes (Wood *et al*., 2009; Renny‐Byfield & Wendel, 2014; Barker *et al*., 2015).

Whilst much has been written on the role of nutrients on plant distribution (Aerts & Chapin, 1999; Craine *et al*., 2002; Harpole *et al*., 2011), the impact of GS has received little attention. In this paper we ask if species with large GS and/or high ploidy level are limited in their productivity by nitrogen (N) and phosphorus (P) nutrient availability. A potential underlying mechanism in limiting angiosperm biomass productivity in terms of GS is that nucleic acids require N and P and larger genomes may be more costly. Phosphorus occurs in plants as inorganic orthophosphate or as organic phosphate esters, of which 40–60% are nucleic acids (Veneklaas *et al*., 2012) and N is another important component of DNA, which has a C : N : P ratio of 9.5 : 3.7 : 1 (Sterner & Elser, 2002). Furthermore N and P are scarce in many unfertilized soils. Phosphorus concentrations in soil range from high (100 μM) to low (1 μM) or very low (0.01 μM), as found in some tropical soils (Johnston *et al*., 2014). By contrast, phosphate concentrations in plant tissues are estimated to range between 5–20 mM, several orders of magnitude higher than soil concentrations (Raghothama, 1999). Up to 80% of soil P is in organic form and must first be mineralised before it is available to plants for uptake, principally in the form of orthophosphate ions (H_2_PO_4_^−^) (Raghothama, 1999). Similarly, N is generally found at concentrations of 1 mM to 0.1 mM; and, as for P, it must first be oxidized or reduced before it is accessible to plants (Novoa & Loomis, 1981). Inorganic phosphate has a low diffusion coefficient in soil and the production of an extensive root system for P scavenging, mining by secretion of carboxylates to solubilise inorganic P, or symbioses with mycorrhizal species are of particular importance in P acquisition (Lambers *et al*., 2008; Richardson *et al*., 2009). By contrast, N is more mobile and its uptake, either as NO_3_^−^ or NH_4_^+^ occurs primarily via a combination of mass flow and diffusion (Richardson *et al*., 2009).

To investigate the impact of N and P on the productivity of species as a function of GS and ploidy, we take advantage of the Park Grass Experiment, Rothamsted, UK, which is the world's longest continuously running ecological experiment, established in 1856 (see the Materials and Methods section). Previously it was shown from this site that fertilizer treatments significantly affected species composition and biomass (Crawley *et al*., 2005). Here we build on that analysis to test the hypothesis that the biomass response to N and P fertilization is dependent on polyploidy level and GS. Specifically, we examine whether N and P availability, and their interaction, differentially affects a species' ability to produce biomass dependent on GS and polyploidy within the competitive setting of a grassland community.

Plant biodiversity and biomass are influenced by both abiotic (e.g. nutrient availability, shade, climate, atmospheric gases) and biotic factors (e.g. soil microorganism communities, competition, predation, pathogens). From the complexity of interactions, Grime (1977) identified three primary plant strategies (the C‐S‐R (competitive, stress‐tolerant, ruderal) plant strategies) that provide both a synthesis of plant responses to environmental stress and predictive power of species occurrence at the community scale. These strategies are defined by the degree to which a species has a propensity to be a competitor for space and resources, is tolerant to environmental stress or is a ruderal, the latter being a species that tends to be short‐lived, weedy and tolerant to disturbance. To investigate the impact of N and P availability on plant biodiversity and biomass, this paper applies Grime's framework to address how ploidy level and GS are associated with C‐S‐R strategies (Grime, 1977) and how these in turn are influenced by N and P availability.

This work directly complements and extends the only other similar study (Šmarda *et al*., 2013) that investigated the effect of P on GS and ploidy of plants growing at the Rengen grassland experiment in Germany, established in 1941. Although Šmarda *et al*.'s study demonstrated that P did indeed influence biomass production, because of the experimental design it was unable to estimate the effects of N and P separately. Here we demonstrate that N and P given separately have no effect on GS and ploidy distributions, but that it is their combined presence that is significant. We also demonstrate that the species that adopt a C strategy at Park Grass are dominated by polyploids with large GS.

Recent work scaling information from local grassland surveys to provide insights into plant distributions over continental scales (Violle *et al*., 2015) has highlighted the need to link our understanding of biodiversity with understanding of ecosystem function and resilience. This is especially important in the face of anthropogenic‐induced stressors such as N and P eutrophication. We propose that GS and ploidy levels might represent important components of biodiversity that can be used to inform such models.

## Materials and Methods

### Study site

The Park Grass Experiment was established in 1856 on 2.8 ha of parkland at Rothamsted in south‐east England, UK, and is the longest continuously running ecological experiment in existence (Lawes *et al*., 1882; Silvertown *et al*., 2006). A detailed overview of the history of fertilizer inputs to the site is given in Crawley *et al*. (2005) and nutrient regimes are summarized in Fig. S2. Briefly, for at least 100 yr before its establishment, the experimental site was natural, unploughed grassland and fertilized occasionally with farmyard manure, road scrapings or guano. One crop of hay was removed annually and a second crop was eaten off by sheep (Lawes & Gilbert, 1859). However, the soil was of poor nutrient status and mildly acidic (pH 5.4–5.6) when the experiment started. The experiment comprises 20 main plots, each receiving specific combinations of mineral fertilizer or organic manures. In 1965 most of the plots were divided into four subplots (a–d), with three (a–c) receiving lime (CaCO_3_), every 3 yr, if necessary, to maintain soil pH levels at 7, 6 and 5. The fourth subplot (d) is unlimed and soil pH here varies from pH 3.6 to pH 5.7 depending on N fertilizer treatment. For consistency, we included only subplots with uninterrupted mineral fertilizer treatments for > 100 yr and a pH > 4.5 in the analyses. In total, we analysed 64 subplots (16 plots and 15 treatment types), including three control plots. Two control plots were established in 1856–1863 and a third, which received farmyard manure from 1856 to 1863, can now be regarded as a control. The herbage on each plot is cut annually in mid‐ to late June and again in autumn. On plots with fertilizer treatment, different combinations of N, P, potassium (K), sodium (Na), magnesium (Mg) and silica (Si) are applied. In terms of N and P nutrients, 11 subplots are treated with N but without P (N subplots), 16 subplots are treated with P but without N (P subplots), 25 subplots get both N and P treatment (N + P subplots), and 12 subplots are control plots (i.e. receive no nutrient treatments). Nitrogen is applied as either (NH_4_)_2_SO_4_ or NaNO_3_. The NH_4_ treatment is applied at four different dosages (nil, low, mid and high) and the NO_3_ is applied at three different dosages (nil, low and mid). These dosages correspond to 0, 48, 96 and 144 kg N ha^–1^ yr^–1^, respectively. Potassium is applied in combination with N on four (out of 11) subplots, eight of the 16 subplots with P, and 19 of the 25 subplots with N + P. Silica is applied on three subplots with N + P. See Fig. S2 for a more detailed description.

### Species at Park Grass

Crawley *et al*. (2005) identified 61 species on the 64 subplots and these are listed in Table S1. All but one are angiosperms, with the remainder being a fern (*Ophioglossum vulgatum*, Monilophyta). Of the angiosperms, 21 are monocots (four families) and 39 eudicots (14 families). *O. vulgatum* occurred on just two subplots with a dry weight (DW) comprising < 1% of subplot herbage yield and was removed from the analyses in order to focus on angiosperms.

All but four species studied at Park Grass are perennial (*Bromus hordeaceus*, annual‐biennial; *Crepis capillaris*, annual‐perennial; *Heracleum sphondylium*, biennial‐perennial; *Tragopogon pratensis*, biennial).

### Biomass, genome size and ploidy estimates

Species DW were taken from Crawley *et al*. (2005) and comprise data obtained from the above‐ground herbage harvested from six small quadrats (50 × 25 cm) within each subplot, before the first cut of the season, from 1991 to 2000. The samples were then sorted into species, oven‐dried and the DW of each species was obtained. The 10 yr mean herbage yields represent the biomass estimates of each species used in this paper.

For GS and ploidy‐level estimations we collected fresh leaves in April–September 2013. We screened 14 taxa (*Achillea millefolium*,* Anthoxanthum odoratum*,* Bromus hordeaceus*,* Briza media*,* Dactylis glomerata*,* Festuca pratensis*,* Festuca rubra*,* Hieracium pilosella*,* Knautia arvensis*,* Lotus corniculatus*,* Lathyrus pratensis*,* Poa pratensis*,* Ranunculus acris*,* Taraxacum officinale*) known to have two or more cytotypes (ploidy level) in Europe. Leaves from at least 12 plants of these species were collected where possible from control plots and from subplots with N + P, and/or midpoint N and P treatment, to examine whether different nutrient regimes selected for different cytotypes. Four taxa (*Ajuga reptans*,* Agrimonia eupatorium*,* Conopodium majus*,* Ononis repens*) without published GS estimates were sampled from plots where they were most common to determine their GS. We also screened three taxa (*Agrostis capillaris*,* Arrhenatherum elatius*,* Holcus lanatus*) for cytotype variation, as these occurred in over 50% of the subplots and across low to high nutrient regimes. Where possible, leaves of the remaining taxa (i.e. taxa with published GS and a single known ploidy level) were sampled in October before the second cut to ensure we were using an appropriate C‐value. GS estimates of taxa which we did not collect were obtained from the Plant DNA C‐values database (Bennett & Leitch, 2012) (Table S2).

1C‐values were estimated by flow cytometry using a Partec CyFlow Space fitted with a Cobalt Samba green (532 nm, 100 mW) laser. Approximately 20 mg of leaf or stem sample was cochopped with either *Petroselinum crispum* ‘Champion Moss Curled’ (1C = 2.22 pg) or *Pisum sativum* ‘Minerva Maple’ (1C = 4.86 pg) as the internal calibration standard in General Purpose Buffer, LB01, Galbraith, Otto or Partec CyStain Absolute P buffer (depending on the species), as described in Pellicer & Leitch (2014). To screen for different ploidy levels within a species, tissues from up to six individual plants were co‐chopped with an internal standard and run on the flow cytometer to measure 1000 or more nuclei. To report GS for *Ajuga reptans*,* Agrimonia eupatorium*,* Conopodium majus* and *Ononis repens*, tissue samples from three individual plants were analysed with each sample run three times, measuring the GS of 5000 or more nuclei per run. The mean coefficient of variation of sample and standard peaks in the flow histograms are reported in Table S2.

### Phylogeny

Evolutionary relationships between species were estimated to account for phylogenetic nonindependence in the statistical analyses. A phylogenetic tree of species found at Park Grass was reconstructed with nucleotide sequences for the plastid markers atpF‐atpH, matK, rbcL, rps16, trnF‐trnL and trnL‐trnT obtained from GenBank (Benson *et al*., 2013) (Table S1), aligned in Mega 5.0 (Tamura *et al*., 2011) using Muscle (Edgar, 2004), checked visually, and concatenated in SeaView (Gouy *et al*., 2010). A maximum likelihood (ML) phylogenetic tree was estimated (Fig. S3) and the position of one family (Caryophyllaceae) was edited in Mega to be consistent with the APG III angiosperm phylogeny (The Angiosperm Phylogeny Group, 2009).

### Statistical analyses

**Figure 1:**
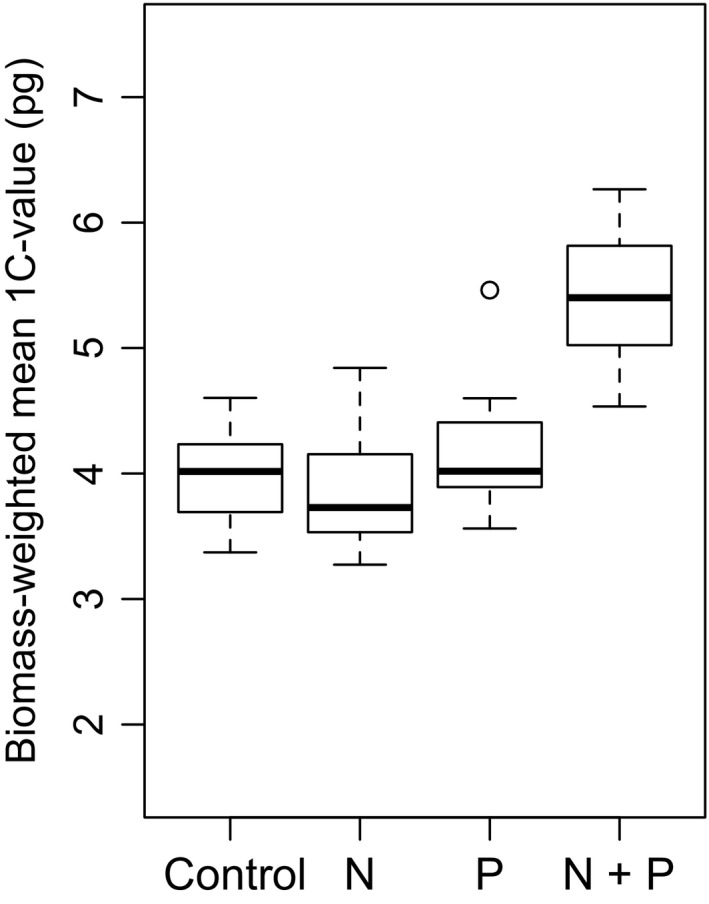
Boxplot showing biomass‐weighted mean 1C‐values of subplots under each of the four fertilizer treatments: control (no N or P added), N (N without P), P (P without N), N + P (both N and P added). Numbers of subplots per treatment are as follows: control, 12 plots; N, 11 plots; P, 16 plots; N + P, 25 plots. See also Supporting Information Table S3 for measures of simple means and SD.

All analyses were carried out in R (R Development Core Team, 2012). We first estimated the biomass‐weighted mean 1C‐value for each of the 64 subplots. This was achieved by summing the product of each species' 1C‐value with its biomass fraction (species subplot biomass/total subplot biomass) (Table 1a). For the boxplot displays (Fig. 1), we grouped the subplot biomass‐weighted mean 1C‐values into one of four categories (control, N, P and N + P), depending on the nutrient treatment. To test the effects of N and P on the biomass‐weighted mean 1C‐values of each subplot in a two‐way ANOVA (Pinheiro *et al*., 2013), we fitted a linear mixed effect (lme) model with the experimental N and P treatments scored as a 2 × 2 factorial N−/N+, P−/P+, with subplot treated as a random effect.

**Table 1:** (a) Means and SD of total biomass, species number, and biomass‐weighted mean 1C‐values of the subplots according to nutrient treatment. (b) Treatment contrasts and ANOVA statistical output testing the effect of nitrogen (N) and phosphorus (P) and their interaction (N : P) on subplot total biomass‐weighted 1C‐values of subplots. The estimated coefficients in the second column show the effects of N application (i.e. without P), the effects of P application (i.e. without N), and the effects when both are applied on a subplot, where the reference level is no application of N or P (i.e. control). (c) Linear mixed effect model significance (*P*‐value) showing the influence of nutrients on biomass‐weighted mean 1C‐value

(a) Treatment	*n*	Total biomass (g)	Species no.	Biomass‐weighted mean 1C‐value (pg)
Control	12	31.71 ± 4.27	39 ± 4.2	3.99 ± 0.37
N	11	34.47 ± 3.9	31 ± 5.6	3.87 ± 0.51
P	16	44.49 ± 11.75	32 ± 4.2	4.17 ± 0.44
N + P	25	58.36 ± 11.64	20 ± 5.5	5.40 ± 0.52

(b)								
	Estimate	Standard error	*t*‐value	Pr(> |t|)	ANOVA	df	*F*‐value	*P*‐value
Intercept	3.984	0.140	28.374	< 0.00001	Intercept	1, 60	5639.792	< 0.0001
N	−0.110	0.203	−0.543	0.58900	**N**	**1, 60**	**47.589**	**< 0.0001**
P	0.164	0.188	0.869	0.38800	**P**	**1, 60**	**46.97**	**< 0.0001**
**N : P**	**1.331**	**0.257**	**5.178**	**< 0.00001**	**N : P**	**1, 60**	**26.816**	**< 0.0001**

(c) Nutrient	*P*‐value
Si	**0.0299**
Na and Mg	0.1367
K	0.9221
**P**	**< 0.0001**
**NO**_**3**_	**< 0.0001**
**NH**_**4**_	**< 0.0001**

Significant parameters are in bold.

We also fitted lme models to investigate the effect of each nutrient applied (i.e. N (NO_3_ and NH_4_), P, K, Si, Na) on biomass‐weighted mean 1C‐value, with subplot treated as random effect. The significance of each nutrient was tested by model reduction with ML inference and the final most parsimonious model was inferred with residual ML.

To examine the impact of ploidy and GS on plant biomass under nutrient limitation at the community level (i.e. individual subplots), we compared total subplot biomass and species numbers of species with large GS (1C ≥ 5 pg) vs species with small GS (1C < 5 pg), and diploids vs polyploids, between the four levels of treatment (control, N, P and N + P; scored as +N/−N, +P/−P). We also present results in the Supporting Information where we set the large GS threshold at ≥ 2.5 pg (i.e. the median GS for angiosperms (Leitch & Leitch, 2013), including for those taxa at Park Grass; 2.53 pg, Fig. S1), ≥ 3 pg and ≥ 6 pg. We also consider GS as a continuous variable (see later). For all thresholds, species were partitioned into four genomic groups: diploid taxa with small GS; diploid taxa with large GS; polyploid taxa with small GS; and polyploid taxa with large GS. We tested to see whether the total biomass of each genomic group was dependent on different N and P treatments in an ANOVA model with interaction terms between GS, ploidy, N and P (subplot was treated as a random factor). Assignment of ploidy level was based on our GS estimations obtained in the present study and chromosome counts published in conjunction with C‐value (Šmarda *et al*., 2007; Bennett & Leitch, 2012; Rice *et al*., 2015). We proceeded to compare biomass‐weighted C‐S‐R strategies between these genomic groups. Biomass and species numbers were square‐rooted to meet model assumptions of normality. C‐S‐R types were attributed to each taxon following Hodgson *et al*. (1999) (Table S2). Each species has a C : S : R ratio that sums to one, the numbers in the ratio being used to partition biomass data. For example, a species with a C‐S‐R category of 0.5 : 0.25 : 0.25 and 10 g of biomass was partitioned with 5 g of biomass to ‘C’ and 2.5 g to each of ‘S’ and ‘R’. To test the significance of GS, ploidy and fertilizer on differential biomass among the C‐S‐R strategies, we performed a multivariate ANOVA with interaction terms between all main effects (i.e. GS, ploidy, N and P, all as binary factors). Because a combination of C‐S‐R strategies was attributed to each species, we did not analyse species numbers for each strategy.

We tested for evidence of phylogenetic signal in the GS data and the C‐S‐R strategies (i.e. nonindependence between phylogeny and e.g. GS) with the function *phylosig* (Revell, 2012). No phylogenetic signal was detected in the C‐S‐R data, but a significant phylogenetic signal was present among species for GS (*K*‐stat = 2.835, *P *=* *0.001, lambda = 1.049). To account for phylogenetic nonindependence, we fitted phylogenetic generalized linear mixed models (pglmm) with Markov chain Monte Carlo techniques (MCMCglmm) (Hadfield, 2010). We tested the effects of GS, ploidy level, N and P treatments and how interactions between these variables contributed to species biomass across all subplots, whilst allowing for phylogenetic correlations. Ploidy, N and P were treated as binary variables. Biomass and 1C‐values were log_e_‐transformed to ensure normality of errors. We treated subplot and species identity as random effects, and phylogeny as a covariance structure (see Methods S1 for phylogenetic tree file). Models were run with five million iterations including a burn‐in of 8000 and a thinning interval of 500, resulting in effective sampling sizes from 9370 to 9984 for all variables and interactions, including random variables. We tested the effect of different priors (e.g. flat (nu = 0), weak (e.g. nu = 0.002, *V* = 1), and expanded priors (alpha.mu = 0, alpha.*V* = 1000)) and found that these had no, or only very small, effects on the estimated means and significance of the parameters. We report here the parameter estimates with a prior where nu = 0.5 and each variance component = 1 as this had the best convergence and chain mixing.

In line with Šmarda *et al*. (2013), we also estimated biomass‐weighted mean 1C‐values using the phylogenetic generalized least‐squares (pgls) method (Paradis *et al*., 2004; Pinheiro *et al*., 2013), which we include in Supporting Information (Fig. S4; Table S3).

## Results

### GS and ploidy diversity of species growing at Park Grass

Genome size ranges 157‐fold amongst the 60 angiosperm species on the plots we analysed, from 0.3 pg in *Carex flacca* to 47.3 pg in *Fritillaria meleagris*, with a median and mean of 2.53 and 4.07 pg, respectively (Fig. S1b). The only taxon we found with a range of GS was *Poa pratensis* (1C‐value = 3.3–7 pg). This species is known to have an extensive variation in chromosome numbers (Rice *et al*., 2015). We used a mean GS of 1C = 4.9 pg for this species (Table S2). We report a new cytotype 1C‐value for *Lathyrus pratensis* at 11.46 pg, the previous reported range being 4.54–7.35 pg (Bennett & Leitch, 2012).

### Subplot biomass‐weighted mean GS

To investigate whether GS contributes to the differential biomass across subplots, we first determined the biomass‐weighted mean 1C‐values for each of four nutrient treatments (control, N, P, N + P) and indicate an increased mean with N + P (Fig. 1; Table 1a). Two‐way ANOVAs and treatment contrasts showed that the subplot biomass‐weighted mean 1C‐value significantly increased only under the addition of both N and P (*F*(1, 60) = 26.82, *P *<* *0.0001, Table 1b). Treatment contrasts showed that the biomass‐weighted mean 1C‐values increased from 3.99 pg on control plots to 5.4 pg on plots with both N and P treatment (Table 1a).

To determine whether these results were primarily the result of the addition of N + P or whether other nutrients added were also having an effect and influencing the results, we used linear mixed effect (lme) models with subplot as a random effect. We found that P, NaNO_3_ and (NH_4_)_2_SO_3_ were highly significantly influencing biomass‐weighted mean 1C‐values (Table 1c). Silica also has a significant effect (*P *=* *0.0299), but it is only applied on one plot with high N + P treatment levels. Given these results, we feel justified in combining the data from the different subplots into the four nutrient categories (i.e. control, N, P and N + P) used earlier.

### Effects of GS, polyploidy, and nutrients on biomass and species numbers

**Figure 2:**
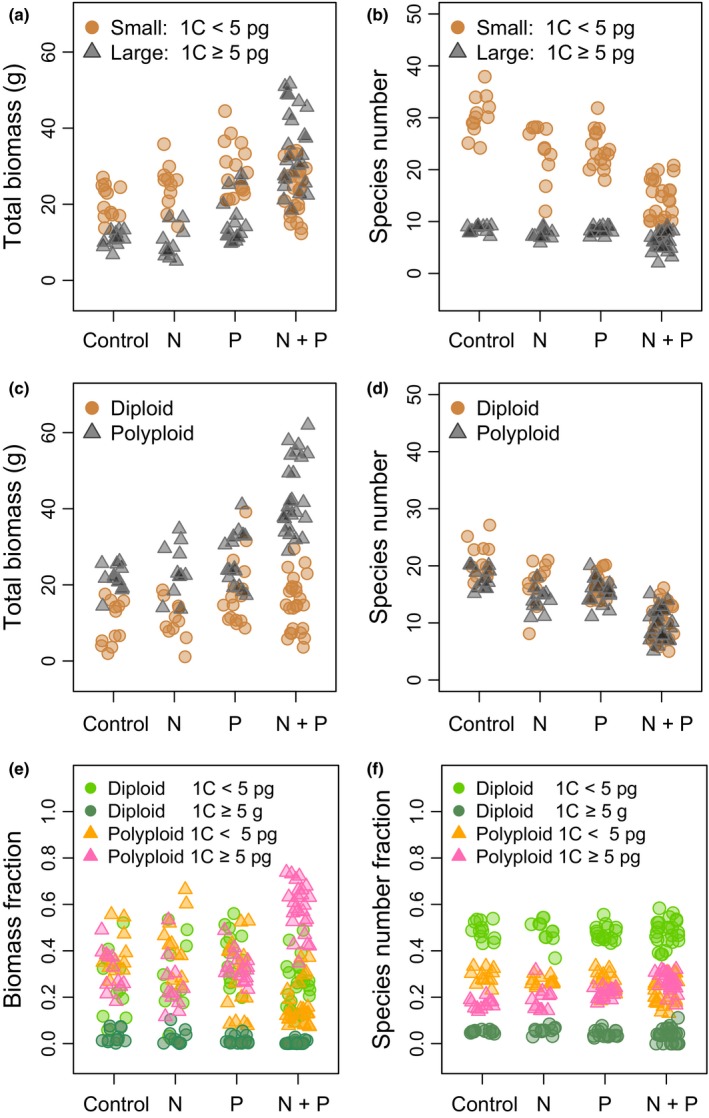
Biomass and species numbers by genomic group. (a–d) Graphs showing the impact of genome size (GS) (small (1C‐value < 5 pg) vs large GS (1C ≥ 5 pg)) (a, b); and the effect of ploidy level (diploid vs polyploidy) (c, d) on total biomass and total number of species, respectively. (e, f) Graphs showing the biomass and species number ratios of the four genomic groups: diploid taxa with small GS, diploid taxa with large GS; polyploids with small GS; and polyploids with large GS. In panels (a)–(d) each subplot is represented by two points, and in (e) and (f) by four points, one for each of the genomic groups. See Tables 1 and Supporting Information Tables S3 and S4 for biomass and species number, ratios, means, SDs; also Figs S5 and S6.

The species biomass data were split into biomass from species with small (1C < 5 pg) and large 1C ≥ 5 pg) GS and diploid and polyploid species (Fig. 2). In terms of simple biomass ratios, plants with small GS comprised around two‐thirds of the total biomass in the control, N and P plots (0.65, 0.72 and 0.66, respectively), whereas in plots with N + P, plants with large GS contributed more than half (0.59) of the total biomass (Table S4a; Fig. 2a). Polyploids dominated biomass under all treatments (0.7, 0.68, 0.61 and 0.75 in control, N, P and N + P treatments, respectively; Fig. 2c; Table S4). ANOVAs show that GS (*F*(1, 180) = 111.17), and ploidy (*F*(1, 180) = 361.88) both have highly significant effects (*P *<* *0.0001) on plant biomass, and that these two genomic parameters interact with N and P (*F*(1, 180) = 11.8, *P *=* *0.0007). Treatment contrasts show that this four‐way interaction (N : P : GS : ploidy) has the largest effect on biomass (Tables S5, S6; see also later). This result is shown visually by splitting the data into four genomic groups (Fig. 2e). The graph shows that increased biomass is associated with polyploids of large GS on N + P plots (mean biomass ratio = 0.584; Table S4a). Across all nutrient treatments, diploid and polyploid species with small GS made similar contributions to biomass, while diploid species with large GS generated little biomass under any treatment (e.g. *Helictotrichon pubescens*,* Ranunculus bulbosus* and *Fritillaria meleagris*) (see Table S4 for means, SDs, and ratios of total biomass and species numbers; Table S5 for ANOVA statistics; Fig. S5 for boxplots of Fig. 2a,c,e; and Fig. S6 for boxplots of Fig. 2b,d,f). Three‐way interactions among P, GS and ploidy level and four‐way interactions involving N as well remain highly significant when different thresholds (1C ≥ 2.5, 3 and 6 pg) are used to delimit large GS (Figs S7–S9; Tables S7–S12).

The total number of species on plots with N + P decreased, as previously reported (Crawley *et al*., 2005). Species diversity in each of the four genomic groups differed significantly (i.e. diploids with large and small GS and polyploids with large or small GS). Treatment contrasts show that GS (*F*(1, 180) = 1719.7, *P *<* *0.0001) and interactions between GS and ploidy ((*F*(1, 180) = 1227.5, *P *<* *0.0001) have the greatest influence on species diversity (threshold for large genome 1C ≥ 5 pg; Figs 2b,d,f, S6; Tables S4, S5). The same was true when using a large GS threshold of ≥ 2.5 and 6 pg. However, for a threshold of ≥ 3 pg, whilst the interaction between GS and ploidy also had the strongest effect on species diversity (*P *<* *0.0001) and GS a significant effect (*P *<* *0.0003), the second strongest influence was N (see Tables S7–S12).

### Testing the impact of C‐S‐R strategies on biomass production under different nutrient regimes

**Figure 3:**
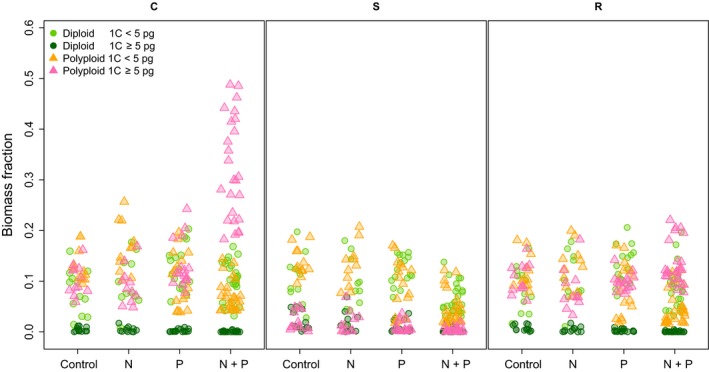
The biomass data shown in Fig. 2 weighted by C‐S‐R strategy (C, competitor; S, stress‐tolerant; R, ruderal). As in Fig. 2(e) and (f), each subplot is represented by four points corresponding to the four genomic parameter groups, with the exception of seven N + P subplots where diploids with 1C ≥ 5 pg were not present and thus are represented by three data points only. See also Supporting Information Table S4.

We investigated whether Grime's (1977) plant strategy categories (i.e. competitors, stress tolerators and ruderals, ‘weeds’) contributed to the distribution of biomass on the Park Grass subplots. It is already known that the addition of fertilizers favours plants with a competitive strategy (Grime, 1977). To determine the effect of these strategies on biomass at Park Grass, we used published C‐S‐R ratios (Hodgson *et al*., 1999) to weight species’ biomass on each subplot (as described earlier for weighting the GS data). We then replotted our data for plants in the four genomic categories (i.e. diploids with large and small GS and polyploids with large or small GS) against plant strategy‐weighted biomass. It was most apparent that N + P subplots were dominated by C strategists, which were polyploids with a large GS (see Fig. 3 and Table S5d) and there were significant interactions among N, P, GS and ploidy (*F*(1, 180) = 16.50, *P *<* *0.0001). These large‐genomed, polyploid C‐strategist species were shown to comprise on average about one‐third of the total biomass of N + P plots (0.32 ± 0.1; Fig. 3; Table S4c) and included two grasses, *A. elatius* and *Alopecurus pratensis*, which contributed as much as 48.77% and 34.75% of biomass, respectively. (Alternative thresholds to define large GS also revealed that the most productive C‐strategists were polyploids with a large GS (Figs S10–S12; Tables S7–S12)). For all thresholds of large GS (Tables S7–S12), the interaction between GS and ploidy had the largest positive effect on biomass productivity of S‐taxa (e.g. 1C ≥ 5 pg, *F*(1, 180) = 349.74, *P *<* *0.0001) and R‐taxa (e.g. 1C ≥ 5 pg, *F*(1, 180) = 504.96, *P *<* *0.0001).

### Testing the effects of GS, ploidy level, and N and P treatment using Bayesian approaches

Whilst the results described earlier are consistent with our assertion that plant strategy, polyploidy and GS interact to influence biomass and species composition, depending on fertilizer input, we also analysed our data using a Bayesian pglmm to address the possibility that results are distorted by the nonindependent response of phylogenetically related species amongst subplots. MCMCglmm confirms that increased biomass involves a significant (*P *=* *0.028; Table 2) three‐way interaction among GS, N and P. Four‐way interactions involving GS, N, P and ploidy are not significant, but three‐way interactions with GS, P and ploidy are significant (*P *<* *0.0001, Table 2). P and pH also have significant positive influences on biomass (Table 2). Interestingly, the two‐way interactions between P and ploidy, and P and GS are significantly negative, meaning that the addition of P without N is associated with small GS diploids increasing their biomass productivity over large GS polyploids. Thus, collectively, these data indicate that biomass production varies not only with fertilizer treatment, but also with its interaction with GS and ploidy level.

**Table 2:** (a) Phylogenetic generalized linear mixed model (MCMCglmm) coefficients (posterior mean), lower and upper 95% credible intervals (CIs) of parameters, the effective sample size taken from the chain, with significant pMCMC values (≤ 0.05 in bold, where pMCMC, used to assess statistical significance, is the probability that the simulated parameters are > 0 or < 0, accounting for the number of MCMC samples): genome size (1C‐value), ploidy, N and P on biomass yield at the Park Grass experiment; (b) statistics are shown for the covariance matrices of the random effects (G‐structure) and the covariance matrix of the residuals (R‐structure)

	Posterior mean	Lower, upper 95% CI	Effective sample size	pMCMC
(a)				
Intercept	−4.573	−6.11, −3.00	9984	< 0.0001
GS	0.186	−0.55, 0.95	9984	0.6202
N	−0.034	−0.57, 0.51	9370	0.905
P	0.907	0.42, 1.39	9984	**< 0.0001**
Ploidy	0.452	−1.01, 1.79	9984	0.5234
pH	0.256	0.12, 0.4	9984	**0.0012**
GS : N	0.04	−0.42, 0.49	9984	0.862
GS : P	−0.536	−0.95, −0.12	9984	**0.013**
N : P	−0.605	−1.37, 0.08	9540	0.098
GS : Ploidy	0.053	−1.05, 1.06	9984	0.9173
N : Ploidy	−0.722	−1.52, 0.03	9984	0.0693
P : Ploidy	−1.411	−2.15, −0.69	9984	**< 0.0001**
GS : N : P	0.71	0.07, 1.34	9984	**0.028**
GS : N : Ploidy	0.503	−0.09, 1.14	9671	0.1078
GS : P : Ploidy	1.225	0.63, 1.78	9984	**< 0.0001**
N : P : Ploidy	−0.872	−2.08, 0.4	9984	0.1735
GS : N : P : Ploidy	0.043	−0.87, 0.96	9984	0.9247
(b)				
G‐structure: ~plot	0.102	0.04, 0.17	9984	
G‐structure: ~phylogeny	0.818	0.04, 2.54	9984	
G‐structure: ~species	2.806	1.62, 4.09	9984	
R‐structure: ~units	3.022	2.83, 3.23	9984	

For completeness and comparisons with Šmarda *et al*. (2013), as an alternative approach to factor in phylogenetic nonindependence of the data, we also analysed the data using the phylogenetic generalized least‐squares (pgls) method (Fig. S4; Tables S3, S6). The output was qualitatively the same as we observed through analyses of biomass‐weighted mean 1C‐value and MCMCglmm outlined earlier.

## Discussion

### Influence of GS on plant biomass under different nutrient inputs

We show that the biomass‐weighted mean 1C‐value and the ploidy level of species growing in the presence of N + P fertilizer are significantly higher than for species on subplots without both these macronutrients (Figs 1, 2). We also show that there is no such response when N and P are added on their own, that is, that the increased biomass from species with large GS and/or polyploidy requires both these nutrients together. The MCMCglmm analysis indicates that GS and ploidy are significant in predicting species biomass dependent on nutrient status.

Soil pH was also shown to influence biomass and this effect may arise through its known impact on nutrient availability. At neutral pH, ammonium (NH_4_^+^) is more rapidly converted to nitrate (NO_3_^−^) by soil microbes, and N fixation by *Rhizobium* in legumes declines with soil acidity. In addition, phosphate forms stable, insoluble minerals and is most available at neutral to slightly acidic pH (Lucas & Davis, 1961; Jensen, 2010). Further, acid conditions can solubilize soil aluminium, which can be toxic to plants, and favour aluminium‐tolerant plant species, including at Park Grass (Gould *et al*., 2014).

As with Park Grass, a similar response to combined N + P was observed in the Rengen grassland experiment in Germany, established in 1941 (Šmarda *et al*., 2013), although that experiment could not dissect the individual impact of N and P, as the experimental design did not include plots where N and P were applied separately. Nevertheless, our second demonstration of the impact of GS and ploidy in influencing biomass growth under different nutrient regimes could point to a general ecological response to N and P availability in the environment. Both experiments have shown that when angiosperms are released from N and P limitation, there is an associated increase in biomass of species with large genomes, a phenomenon associated with increased biomass generated by polyploid taxa (see later). These results agree with observations showing that the combined input of N and P into terrestrial, aquatic, and marine environments produces much stronger responses in plant community biomass production than N and P alone (Elser *et al*., 2007; Harpole *et al*., 2011), although such studies did not investigate the impact of GS and ploidy levels.

The requirements of N and P are clearly interlinked even though the properties of these two elements differ. Cellular processes such as transcription and translation require a coupling of N and P, where P is needed for mRNA synthesis, followed by translation, which requires N for protein production. An increase in N facilitates the production of phosphatase enzymes that cleave ester‐P bonds in soil to increase rates of P uptake (Vitousek *et al*., 2010; Marklein & Houlton, 2012), while the availability of P is known to influence the rates of N fixation or denitrification (Sterner & Elser, 2002). At the genomic level, transcription factors that suppress primary root growth may be regulated by both N and P (Medici *et al*., 2015). Furthermore, while photosynthetic capacity has often been shown to be related to leaf N concentrations, such a relationship is constrained in P‐limited environments, possibly as a result of limitations of ribulose‐1,5‐bisphosphate or ribulose‐1,5‐bisphosphate carboxylase (RuBisCO) regeneration in plants which are P‐deficient (Reich *et al*., 2009). Taken together, such interactions may well contribute to explaining why it is only when N and P are added together that a significant increase in biomass is observed.

However, it is also necessary to explain the significant impact of GS and ploidy in influencing the plant response to N and P. Currently, how the large 2400‐fold range in angiosperm GS influences N and P demands in the plant is unknown, yet GS is likely to have significant resource implications because nucleic acids are amongst the most N‐ and P‐demanding biomolecules of the cell, being *c*. 39% N and nearly 9% P by mass. DNA must also be packaged in the nucleus, which requires N‐demanding histones, one of the most abundant proteins of the cell (Sterner & Elser, 2002). That N demand may also lead to tradeoffs for N with RuBisCO.

Given our observation that N and P impact species composition dependent on GS and ploidy, it suggests that DNA is demanding for these nutrients. However, it is also known that cell size (Hodgson *et al*., 2010) and other factors (e.g. growth rates, cell division time; see review in Greilhuber & Leitch, 2013) correlate with GS across the range of GS found in angiosperms. Indeed, at Park Grass, guard cell size correlates with GS (Fig. S13). Potentially, increased N and P demands associated with GS at a cellular level could be offset by a reduction in the total number of cells in a tissue or overall, leading to altered metabolism, growth rates or RNA abundance (Coate & Doyle, 2015). As far as we are aware, no data on total plant N and P associated with C‐value have yet been obtained. To calculate that total is nontrivial, as it requires knowledge of the N and P loading in all tissues (roots, stems, leaves), the biomass of these tissues, and will vary with macronutrient availability in the soil and ontogenetic stage of the plant. Such calculations would best be derived under limiting nutrient conditions, to offset against N and P storage systems. To add further complications, because GS correlates with cell size, there may also be additional effects on photosynthesis efficiency, because increased cell size alters the dynamics of gas exchange in the leaf (Drake *et al*., 2013). Collectively, increases in GS probably lead to tradeoffs in resource allocation between cellular compartments, resulting in altered growth parameters, life strategies and ecology under different nutrient regimes.

### Selection against polyploids in limited‐nutrient conditions

Angiosperm evolution is associated with multiple rounds of polyploidy; indeed, even apparently diploid species are now considered to be paleopolyploids (Van de Peer *et al*., 2009; Jiao *et al*., 2011; Renny‐Byfield & Wendel, 2014). Allopolyploids (produced by interspecific hybridization, and genome doubling) may benefit from hybrid vigour and fixed heterozygosity (Chen, 2010), and the evolution of novel ‘transgressive’ characters (Rieseberg & Willis, 2007). Furthermore, the duplicated gene copies in polyploids are freed from selective constraints, potentially enabling the evolution of new functions (Soltis & Soltis, 2000; Soltis *et al*., 2009). Polyploids are often associated with broader ecological niches and/or invasiveness, leading to greater evolutionary success than diploids (Hegarty & Hiscock, 2008; te Beest *et al*., 2012). Certainly, this combination of advantageous characters may contribute to explaining why polyploid taxa with large GS dominate total biomass under high nutrient (N + P) conditions, especially in contrast to diploid taxa with large GS (Fig. 2e). Overall, the higher biomass of polyploids at Park Grass suggests a disparity in productivity between diploids and polyploids. However, diploids and polyploids with a GS < 5 pg have similar biomass (Fig. 3) and the shift in biomass ratios of polyploid : diploid taxa from *c*. 2 : 1 on low nutrient and control plots to *c*. 3 : 1 on N + P plots suggests that ploidy level alone is not sufficient to determine what constitutes a highly successful polyploid. Instead, the observed distribution and abundance of different plant species at Park Grass are the result of more complex processes influenced by effects of GS on growth and competitiveness of polyploids, both mediated through interactions with N and P. We also observed a significant increase in biomass of diploid plants with a small genome associated with the application of P without N (Table 2), perhaps because these plants are less N‐demanding and can better utilize available P. Thus paramount amongst the costs of high GS polyploids could be the increased biochemical demand for cellular N and P generated by GS multiplication. These costs should be considered alongside the more widely acknowledged costs associated with polyploidy, such as minority cytotype exclusion (Otto, 2007) and chromosome pairing problems in meiosis (Comai, 2005), which can also lead to polyploids having lower fitness compared with diploid taxa (Burton & Husband, 2000).

These data are consistent with the hypothesis that polyploids with large GS are demanding of N and P. Potentially, the increased nuclear demands for N and P could be offset by altering the total volume of RNA in the transcriptome. For example, it is known that the ‘genomic shock’ generated by *de novo* polyploidy results in plants with variable transcriptome volumes (Grover *et al*., 2012). Selection under limiting N and/or P could favour RNA‐efficient variants with smaller total transcriptome volumes and/or RNA transcripts that are less N‐demanding (Acquisti *et al*., 2009a,b). Nevertheless, the increased biomass of polyploid species on subplots receiving N + P (especially those with large genomes) suggests that polyploids on other subplots at Park Grass are under nutrient limitation.

### Competitor taxa are predominantly polyploids with large genomes

Plants are typically limited by multiple resources, including competition for nutrients, space and light, and have evolved strategies to overcome these limitations. Of these, species adopting competitive strategies (C‐taxa), as described by Grime's C‐S‐R strategies (Grime, 1977), were expected to dominate on high nutrient plots. Indeed, this is what we observed at Park Grass, but in addition we see that the C‐taxa dominating the N + P subplots tend to be polyploids with large (1C ≥ 5 pg) genomes (Fig. 3). Of these species, those that are also found on control or low‐nutrient plots produce only limited biomass and show no competitive advantage (e.g. *A. elatius* and *A. pratensis*).

We suggest that there may be an upper threshold in GS for species with a competitive growth strategy, as we suspect that species with very large GS (1C > 35 pg; as defined in Leitch *et al*., 1998) are predominantly stress‐tolerant (S‐taxa), limited to a slow‐growing, long‐lived life history. This hypothesis needs to be formally tested. From our data, the only species at Park Grass with a very large GS was *Fritillaria meleagris*, a slow‐growing bulbous diploid with a 1C‐value of 47.3 pg (more than four times larger than the next largest GS at Park Grass). While one might expect this species to thrive in subplots with N + P because of the high N and P demands for maintaining such a large genome, it was only found in subplots with just N, suggesting that it is unable to compete with the fast‐growing C‐taxa when both N and P are present in the subplots. Instead, this species is probably limited by factors other than nutrient availability, and perhaps this has led to drift in its GS to its current astonishingly large size. GS itself may now constrain the adaptive potential of *F. meleagris*, because of the effect of GS on cell division rates (Bennett, 1972; Cavalier‐Smith, 2005; Knight *et al*., 2005; Greilhuber & Leitch, 2013; Veselý *et al*., 2013).

### Global scope and scaling to landscape levels

Our data show that plants respond differently to environmental availability of both N and P, depending on GS and ploidy. If the results reported here, together with those of Šmarda *et al*. (2013), are generalities, then there are significant ecological implications to our understanding of plant assemblages, distributions and occurrences. Potentially the patterns we observe occur at multiple scales up to, and including, continental scales, all of which are influenced by, for example, underlying geologies, soil types, soil age, soil pH and farming practices, and all with their own N and P dynamics. Those dynamics will provide selective pressures on species and their evolution, shaping species communities. It has been shown that trait‐based studies can be used in models aiming to link community structures with ecosystem functioning (Violle *et al*., 2015). In addition, it has been suggested that GS is a trait that could be incorporated into such models (Suda *et al*., 2015). Because the Park Grass Experiment is the longest continuously running field trial, we are able to detect significant measurable impacts of GS and ploidy on community structure depending on nutrient availability. We recommend that the Plant DNA C‐values database (http://data.kew.org/cvalues/) is integrated into the TRY Plant Trait database (https://www.try-db.org/TryWeb/Home.php) to facilitate future studies. We anticipate that this will improve the predictive power of models and will enable us to determine more accurately the role played by GS in community structure and ecosystem functioning.

## Author contributions

M.S.G., I.J.L., A.R.L. A.M. and M.T. planned and designed research; M.S.G. conducted fieldwork; M.S.G. and C‐A.R. performed laboratory work; R.A.N. and R.J.K. provided statistical expertise and with M.S.G. statistically analysed the data. M.S.G., A.R.L., and I.J.L. took the lead with writing the manuscript, with significant input from M.T., R.A.N, R.J.K. and A.M. All authors revised, read and approved the final manuscript.

## Supporting information

Please note: Wiley Blackwell are not responsible for the content or functionality of any supporting information supplied by the authors. Any queries (other than missing material) should be directed to the *New Phytologist* Central Office.

**Fig. S1** Histogram of (a) available angiosperm genome size (GS) data, and (b) GS of species present in the Park Grass data.
**Fig. S2** Diagram showing plot layout at the Park Grass Experiment with fertilizer treatments and plots sampled.
**Fig. S3** Phylogenetic tree of 60 angiosperm species present at the Park Grass experimental plots with GS and ploidy level.
**Fig. S4** Boxplots showing phylogenetic generalized‐least squares (pgls) biomass‐weighted mean 1C‐values.
**Fig. S5** Boxplots of Fig. 2, showing the mean total biomass of each genomic group: diploid taxa with small GS; diploid taxa with large GS; polyploids with small GS; and polyploids with large GS.
**Fig. S6** Boxplots of Fig. 2, showing number of species in each genomic group: diploid taxa with small GS; diploid taxa with large GS; polyploids with small GS; and polyploids with large GS.
**Figs S7–S12** The following figures show results of using three different thresholds (in addition to the 5 pg threshold in the main text) when defining large GS: 2.5, 3, and 6 pg.
**Fig. S13** Scatter plot showing phylogeny‐independent contrasts (PIC) on log_10_ mean guard cell length and log_10_ 1C‐value in 27 taxa collected from the Park Grass Experiment plots.
**Table S1** List of and accessions obtained from NCBI's GenBank to estimate phylogenetic relationships
**Table S2** Taxa list with abbreviations, GS, ploidy, flow cytometry GS estimations, and allocated C‐S‐R strategy
**Table S3** Subplot summary: total biomass, number of species, mean GS, including mean *pgl*s GS under control, N, P and N + P fertilizer treatments
**Table S4** Biomass, species numbers, and C‐S‐R‐weighted biomass of the four genomic groups, where large GS ≥ 5 pg
**Table S5** ANOVA results testing significance of GS, ploidy, and fertilizer on biomass, species number, and C‐S‐R weighted biomass, where large GS ≥ 5 pg
**Table S6** ANOVA results testing N and P significance on mean pgls GS under control, N, P and N + P fertilizer treatments
**Tables S7–S12** Summary statistics and ANOVA results from analyses using three different thresholds when defining large GS: 2.5, 3, and 6 pg
**Methods S1** Phylogenetic tree in Newick format used in pgls and phylogenetic generalised mixed model analyses.

**Methods S2** Park Grass species biomass data in CSV format.
