# Supplementary material for: Genome size and ploidy influence angiosperm species' biomass under nitrogen and phosphorus limitation
Source: New Phytol. 2016 Feb 15;210(4):1195–206. doi: 10.1111/nph.13881 (PMC4991274; doi:10.1111/nph.13881)
Supplement: Supplementary file 1 — Fig. S1 Histogram of (a) available angiosperm genome size (GS) data, and (b) GS of species present in the Park Grass data. Fig. S2 Diagram showing plot layout at the Park Grass Experiment with fertilizer treatments and plots sampled. Fig. S3 Phylogenetic tree of 60 angiosperm species present at the Park Grass experimental plots with GS and ploidy level. Fig. S4 Boxplots showing phylogenetic generalized‐least squares (pgls) biomass‐weighted mean 1C‐values. Fig. S5 Boxplots of Fig. 2, showing the mean total biomass of each genomic group: diploid taxa with small GS; diploid taxa with large GS; polyploids with small GS; and polyploids with large GS. Fig. S6 Boxplots of Fig. 2, showing number of species in each genomic group: diploid taxa with small GS; diploid taxa with large GS; polyploids with small GS; and polyploids with large GS. Figs S7–S12 The following figures show results of using three different thresholds (in addition to the 5 pg threshold in the main text) when defining large GS: 2.5, 3, and 6 pg. Fig. S13 Scatter plot showing phylogeny‐independent contrasts (PIC) on log10 mean guard cell length and log10 1C‐value in 27 taxa collected from the Park Grass Experiment plots. Table S1 List of and accessions obtained from NCBI's GenBank to estimate phylogenetic relationships Table S2 Taxa list with abbreviations, GS, ploidy, flow cytometry GS estimations, and allocated C‐S‐R strategy Table S3 Subplot summary: total biomass, number of species, mean GS, including mean pgls GS under control, N, P and N + P fertilizer treatments Table S4 Biomass, species numbers, and C‐S‐R‐weighted biomass of the four genomic groups, where large GS ≥ 5 pg Table S5 ANOVA results testing significance of GS, ploidy, and fertilizer on biomass, species number, and C‐S‐R weighted biomass, where large GS ≥ 5 pg Table S6 ANOVA results testing N and P significance on mean pgls GS under control, N, P and N + P fertilizer treatments Tables S7–S12 Summary statistics and ANOVA results from analys [file NPH-210-1195-s001.pdf]

New Phytologist

New Phytologist **Supporting Information Figs S1–S13, Tables S1–S12 and Methods S1**

Article title: Genome size and ploidy influence angiosperm species biomass under nitrogen and phosphorus limitation

Authors: Maïté S. Guignard, Richard A. Nichols, Robert J. Knell, Andy Macdonald, Mark Trimmer, Catalina-Andreea Romila, Ilia J. Leitch, and Andrew R. Leitch

Article acceptance date: 21 December 2015

The following Supporting Information is available for this article:

Fig. S1 Histogram of (a) available angiosperm genome size (GS) data, and (b) GS of species present in the Park Grass data.

Fig. S2 Diagram showing plot layout at the Park Grass Experiment with fertilizer treatments and plots sampled.

Fig. S3 Phylogenetic tree of 60 angiosperm species present at the Park Grass experimental plots with GS and ploidy level.

Fig. S4 Boxplots showing phylogenetic generalized-least squares (*pgls*) biomass-weighted mean 1C-values.

Fig. S5 Boxplots of Fig. 2, showing the mean total biomass of each genomic group: diploid taxa with small GS, diploid taxa with large GS, polyploids with small GS; and polyploids with large GS.

Fig. S6 Boxplots of Fig. 2, showing number of species in each genomic group: diploid taxa with small GS, diploid taxa with large GS, polyploids with small GS; and polyploids with large GS.

Figs S7–S12 The following figures show results of using three different thresholds (in addition to the 5pg threshold in the main text) when defining large GS: 2.5, 3, and 6 pg.

Fig. S13 Scatter plot showing phylogeny-independent contrasts (PIC) on $\log_{10}$ mean guard cell length and $\log_{10}$ 1C-value in 27 taxa collected from the Park Grass Experiment plots.

Table S1 List of and accessions obtained from NCBI's GenBank to estimate phylogenetic relationships

Table S2 Taxa list with abbreviations, GS, ploidy, flow cytometry GS estimations, and allocated C-S-R strategy

Table S3 Subplot summary: total biomass, number of species, mean GS, including mean *pgl*s GS under control, N, P and N + P fertilizer treatments

Table S4 Biomass, species numbers, and C-S-R-weighted biomass of the four genomic groups, where large GS ≥ 5pg

Table S5 ANOVA results testing significance of GS, ploidy, and fertilizer on biomass, species number, and C-S-R weighted biomass, where large GS ≥ 5pg

Table S6 ANOVA results testing N and P significance on mean *pgls* GS under control, N, P and N + P fertilizer treatments

Tables S7–S12 Summary statistics and ANOVA results from analyses using three different thresholds when defining large GS: 2.5, 3, and 6 pg

Methods S1 Phylogenetic tree in Newick format used in *pgls* and phylogenetic generalised mixed model analyses.

Methods S2 Park Grass species biomass data in CSV format (separate CSV file).

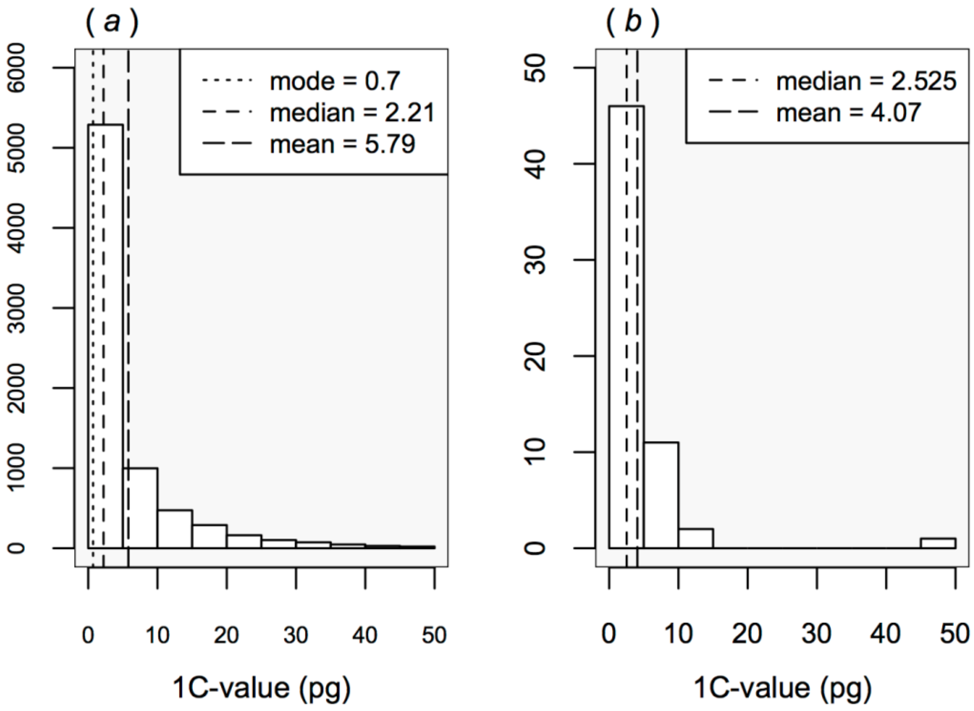

Fig. S1 Distribution of GS for (a) 7,484 angiosperms across 248 families, for species with 1C-values $\leq$ 50 pg. The mode, median and mean 1C-values (pg) for all 7,542 angiosperms listed in Bennett & Leitch (2012) are shown; (b) the 60 angiosperm species from 18 families on Park Grass, which range from 1C = 0.30 pg in *Carex flacca* to 1C = 47.3 pg in *Fritillaria meleagris*. Genome size data in (a) were obtained from: **Bennett MD, Leitch IJ. 2012.** *Plant DNA C-values database, release 6.0, December 2012*. URL http://data.kew.org/cvalues.

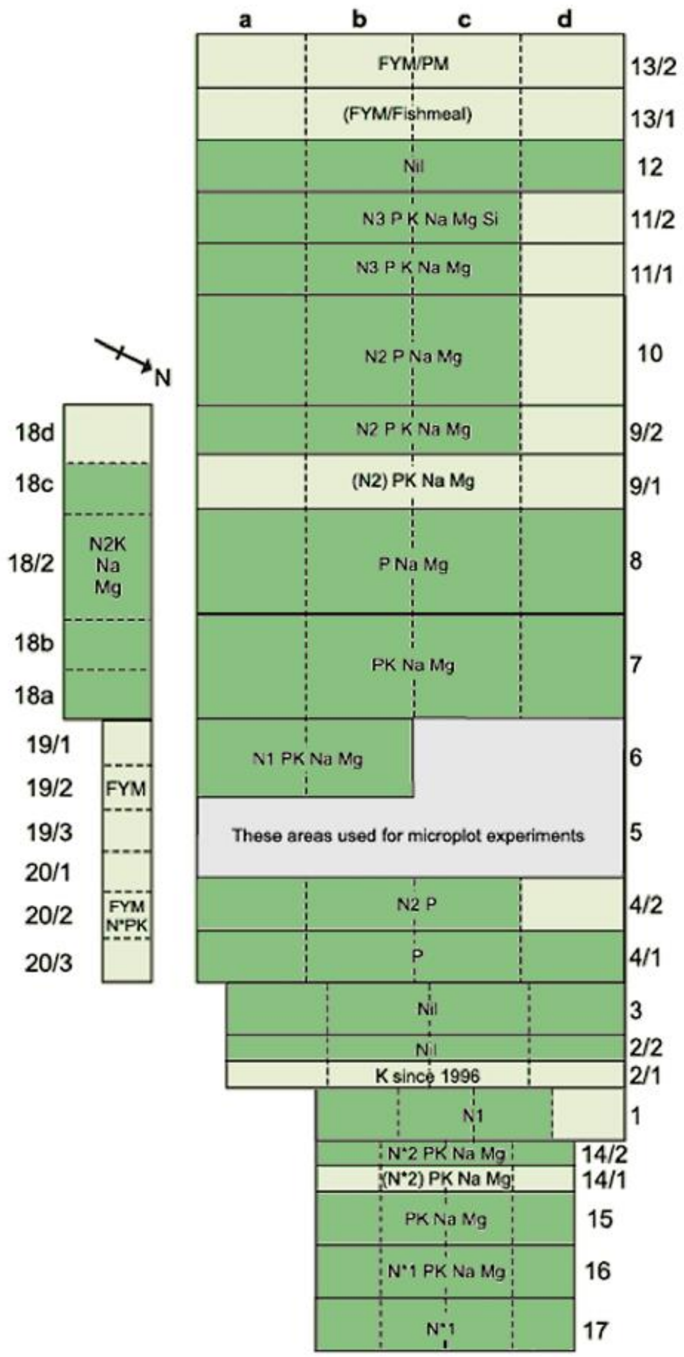

Fig. S2 Plot layout at Park Grass Experiment with fertilizer treatments (as it was when the herbage samples used in this paper were collected). We sampled 64 subplots, highlighted in dark green; these are subplots where macronutrient treatments have remained constant for at least a century. On these subplots, 15 combinations of N, P, K, Na, Mg, and Si are applied. Abbreviations are as follows: N,

$(NH_4)_2SO_4$; N*, $NaNO_3$; where 1, 2 and 3 correspond to 48, 96 and 144 kg of N applied ha^{-1} yr^{-1}, respectively. The other nutrients are applied annually at the following dosages: P, 35 kg ha^{-1}; K, 225 kg ha^{-1}; Na, 15 kg ha^{-1}; Mg, 10 kg ha^{-1} (Na and Mg always added together); and Si, 450 kg ha^{-1}. Abbreviations are as follows: P, P_2O_5; K, K_2SO_4; Mg, $MgSO_4$; Na, Na_2SO_4, Si, Na_2O_3Si. Two control plots receiving no nutrient treatments were established in 1856 (plots 3 and 12) and a third one (plot 2/2) was established in 1864. In 1903 most of the plots were divided into two to test the effects of lime ($CaCO_3$, 4 t ha^{-1}) applied every 4 yr to the southern halves. In 1965 the plots were divided again into four smaller subplots (a–d), with subplots (a), (b) and (c) receiving lime, every 3 yr, to maintain the soil pH at 7, 6, and 5 respectively. The fourth subplot (d) remains unlimed and soil pH here varies from pH 3.6 (on subplots receiving N as $(NH_4)_2SO_4$) to pH 5.7 (on subplots receiving N as $NaNO_3$); control plots (receiving no treatments) are at *c*. pH 5.1. The herbage on each plot is cut annually in mid–late June and again in autumn. The plots were originally cut by scythe, then by horse-drawn and then tractor-drawn mowers. Yields were originally estimated by weighing the produce from the whole plot, either as hay (1st harvest) or green crop (2nd harvest), and dry matter determined. Since 1960, yields of dry matter have been estimated from strips cut with a forage harvester. However, for the first cut the remainder of each plot is still mown and made into hay, continuing earlier management and ensuring the return of seed. For the second cut, the whole of each plot is cut with a forage harvester. For more information on the Park Grass Experiment and recent changes in fertilizer treatments, see http://www.era.rothamsted.ac.uk/index.php?area=home&page=index&dataset=1.

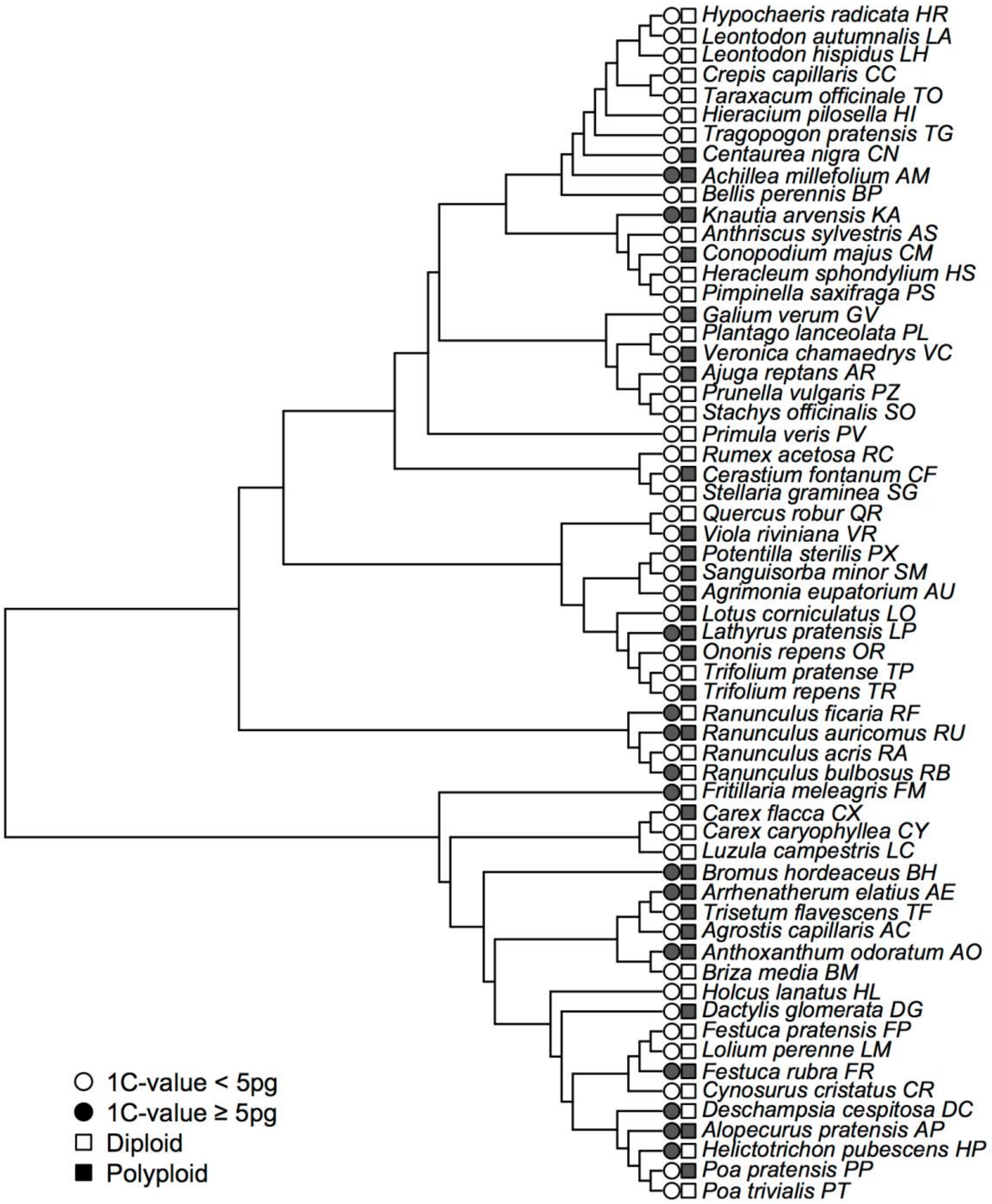

Fig. S3 Maximum likelihood phylogenetic tree of 60 angiosperm species present at the Park Grass experimental plots which was used to obtain a covariance matrix for fitting linear regressions, phylogenetic independent contrasts and phylogenetic generalised linear mixed effect models. Taxa abbreviations are given next to the full taxa name. Taxon markers show whether the 1C-value < 5 pg, or ≥ 5 pg; and whether a taxon is diploid or polyploid. See also Methods S1.

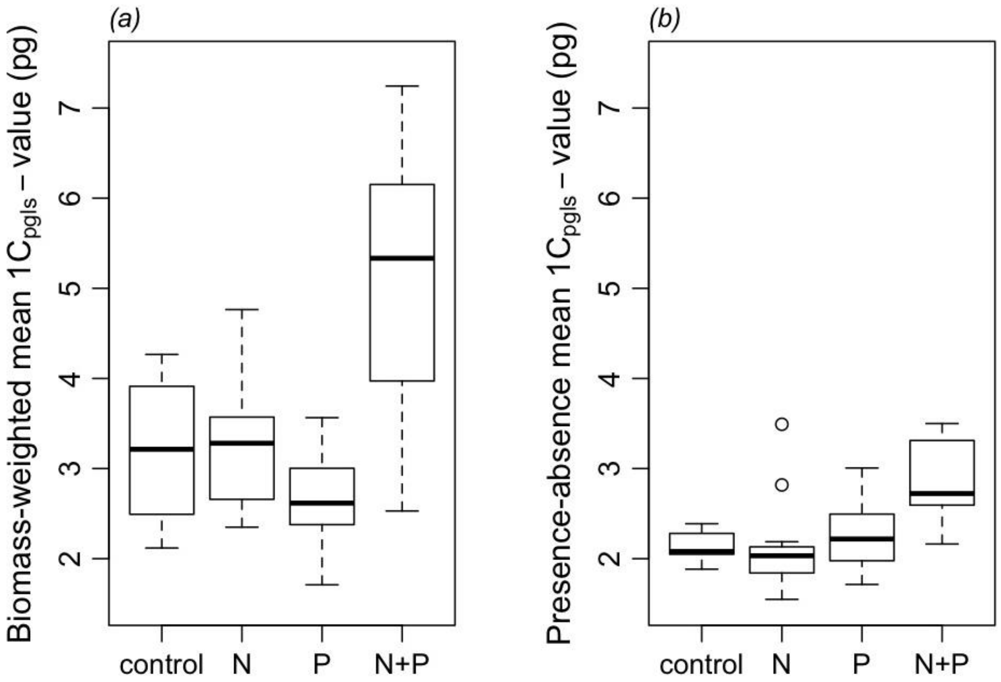

Fig. S4 Boxplots showing (a) biomass-weighted mean 1C$_{pgls}$-values (i.e. phylogeny-adjusted GS weighted by species biomass); (b) presence-absence mean 1C$_{pgls}$-values (i.e. phylogeny-adjusted GS unweighted by biomass). See also legend to Table S4. pgls, phylogenetic generalised least squares.

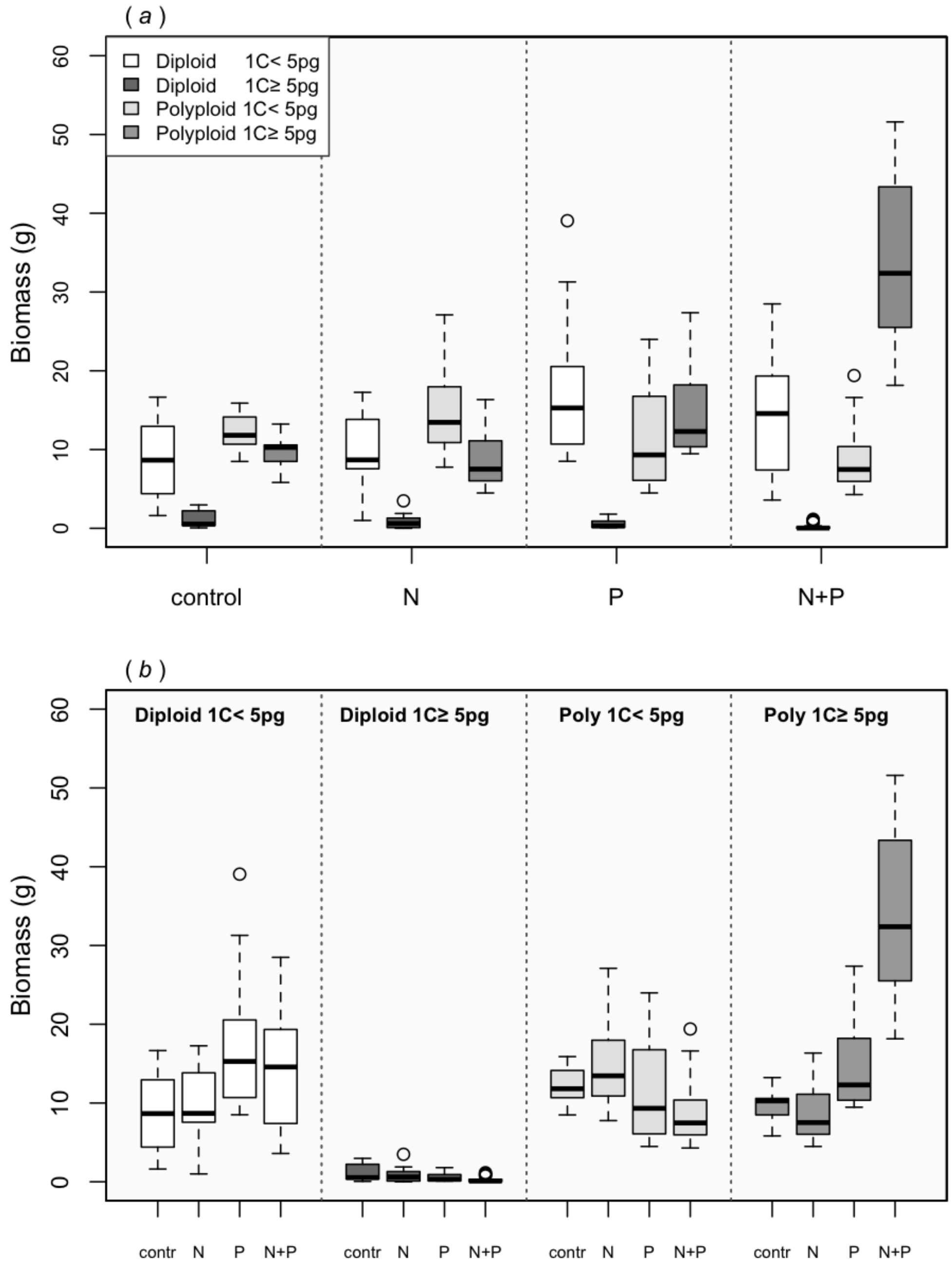

Fig. S5 Boxplots are shown here to facilitate comparison between the four genomic groups of: diploid taxa with small GS (1C-value < 5 pg); diploid taxa with large GS (1C-value ≥ 5 pg); polyploids with small GS; and polyploids with large GS; their mean total biomass is shown in boxplots by (a) treatment, and (b) by genomic group. contr, control (i.e. no nutrients); Poly, polyploids.

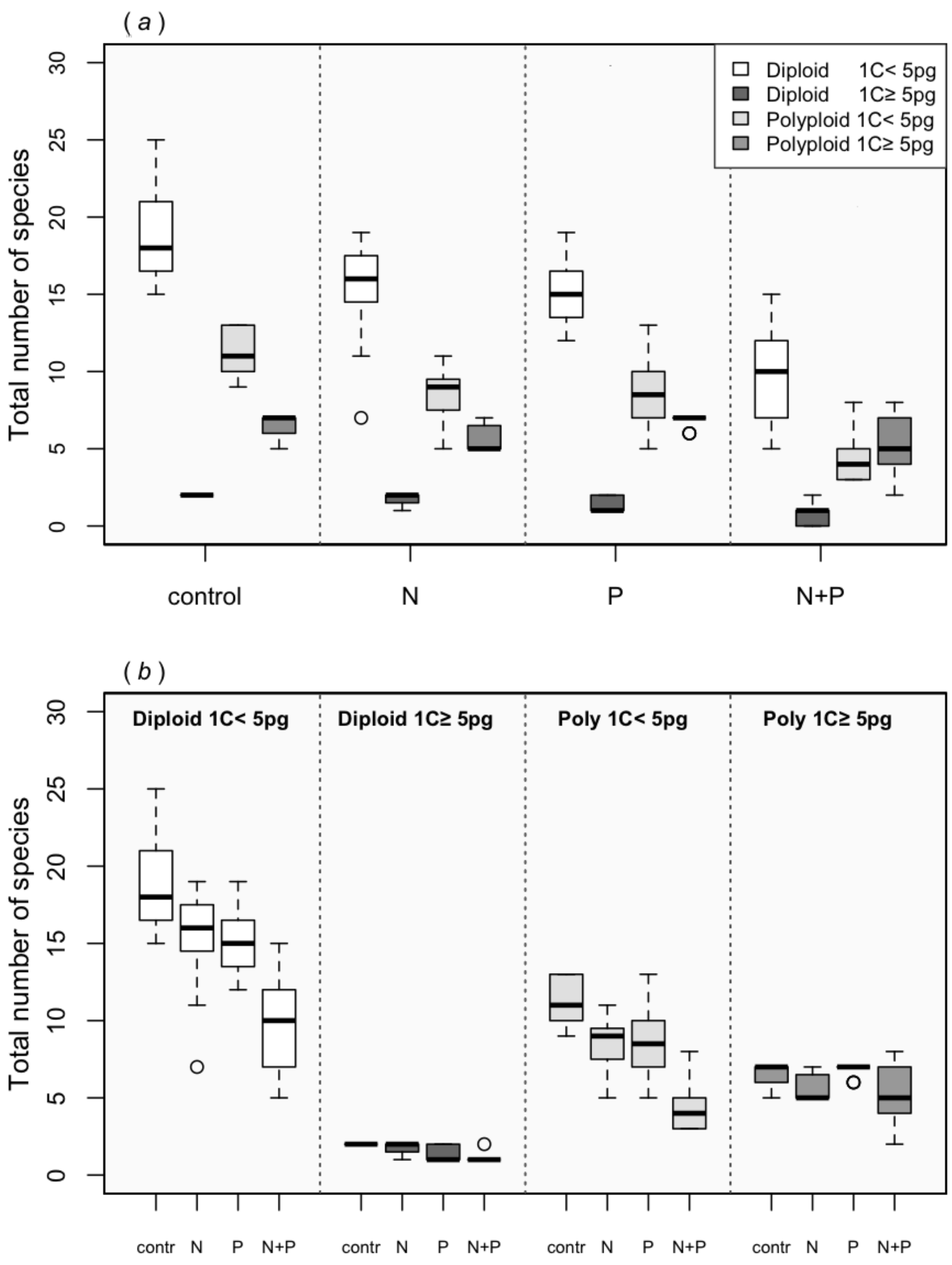

Fig. S6 Boxplots show the comparisons between the four genomic groups of: diploid taxa with small GS (1C-value < 5 pg); diploid taxa with large GS (1C-value ≥ 5 pg); polyploids with small GS; and polyploids with large GS; the total number of species is shown in boxplots by (a) treatment, and (b) by genomic group. contr, control (i.e. no nutrients); Poly, polyploids.

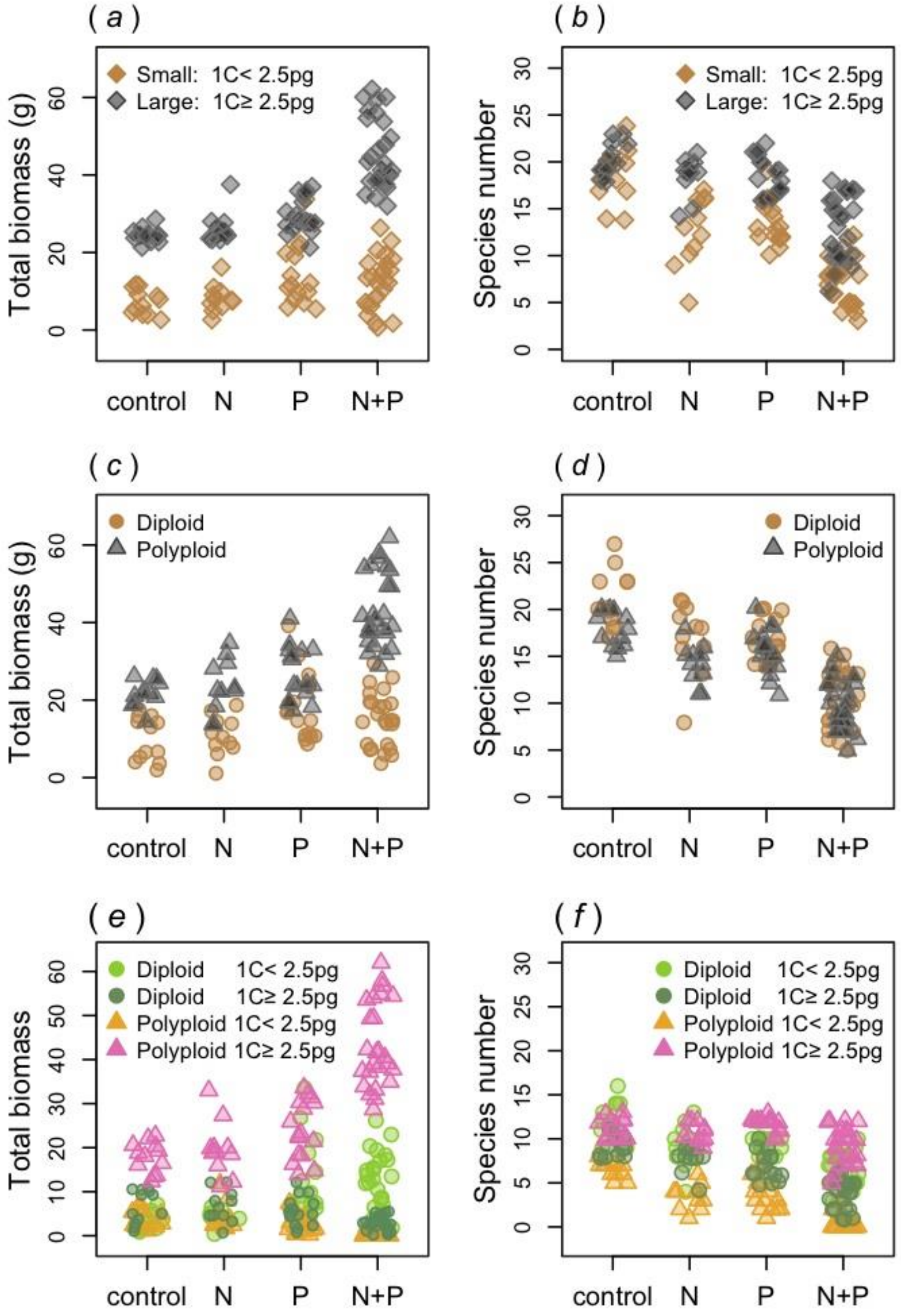

Fig. S7 Large GS ≥ 2.5 pg. Scatter plots comparing total biomass and species numbers between taxa with (a, b) small vs large GS; (c, d) diploid vs polyploid taxa; and (e, f) the four groups based on GS and ploidy level: diploid taxa with small GS (1C-value < 2.5 pg); diploid taxa with large GS (1C-value ≥ 2.5 pg); polyploids with small GS; and polyploids with large GS. See also Tables S7 and S8.

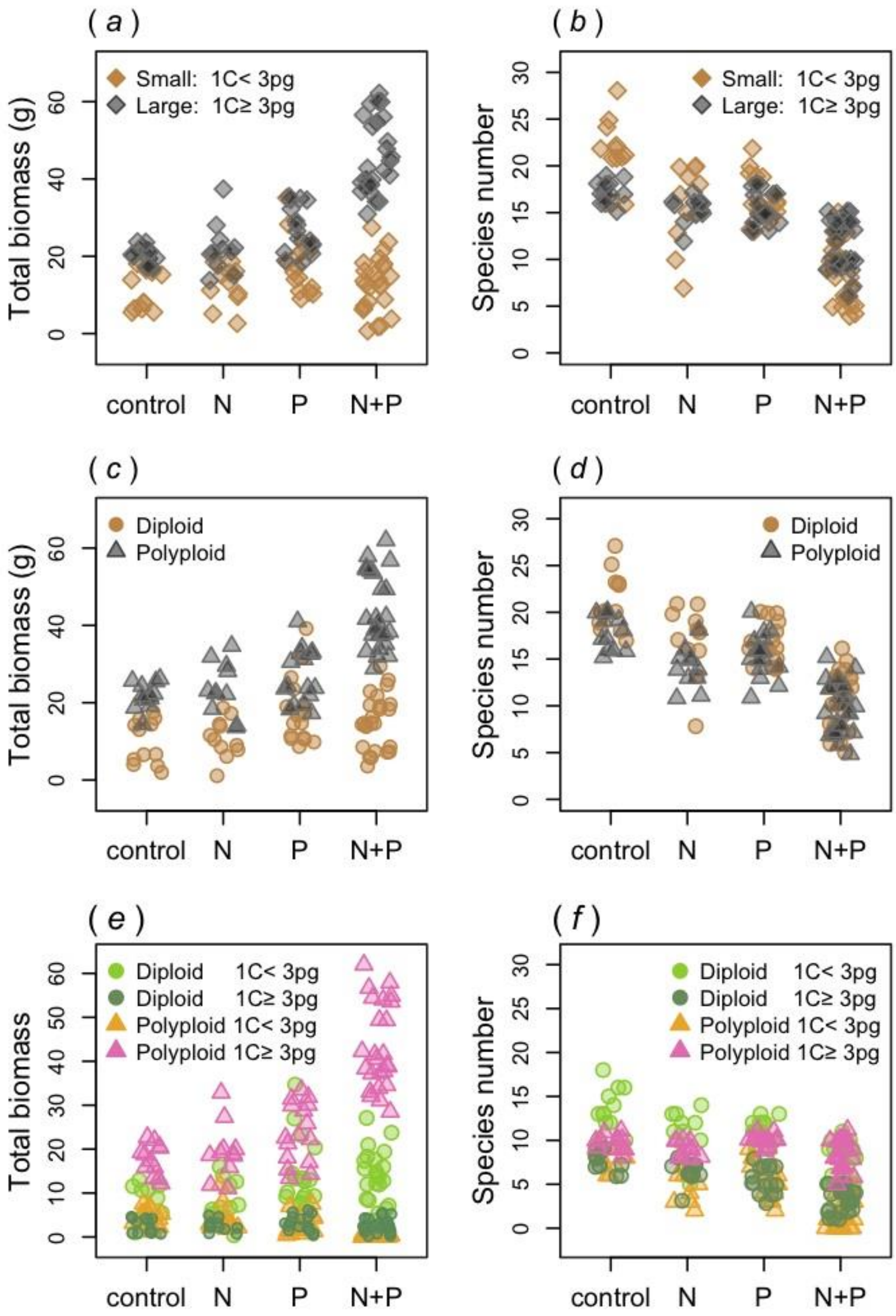

Fig. S8 Large GS ≥ 3 pg. Scatter plots comparing total biomass and species numbers between taxa with (a), (b) small vs large GS; (c), (d) diploid vs polyploid taxa; and (e), (f) the four groups based on GS and ploidy level: diploid taxa with small GS (1C-value < 3 pg); diploid taxa with large GS (1C-value ≥ 3 pg); polyploids with small GS; and polyploids with large GS. See also Tables S9 and S10.

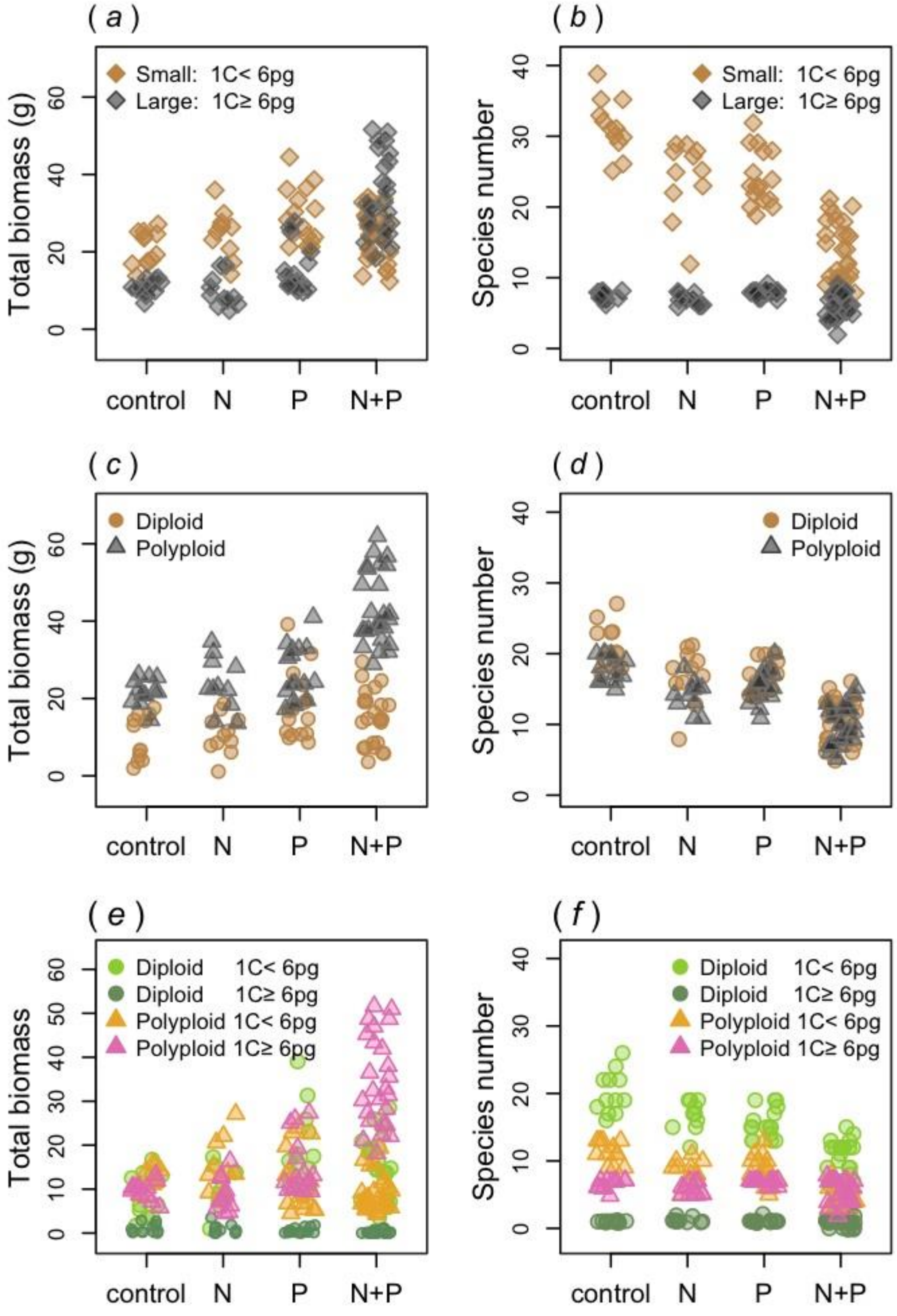

Fig. S9 Large GS ≥ 6 pg. Scatter plots comparing total biomass and species numbers between taxa with (a, b) small vs large GS; (c, d) diploid vs polyploid taxa; and (e, f) the four groups based on GS and ploidy level: diploid taxa with small GS (1C-value < 6 pg); diploid taxa with large GS (1C-value ≥ 6 pg); polyploids with small GS; and polyploids with large GS. See also Tables S11 and S12.

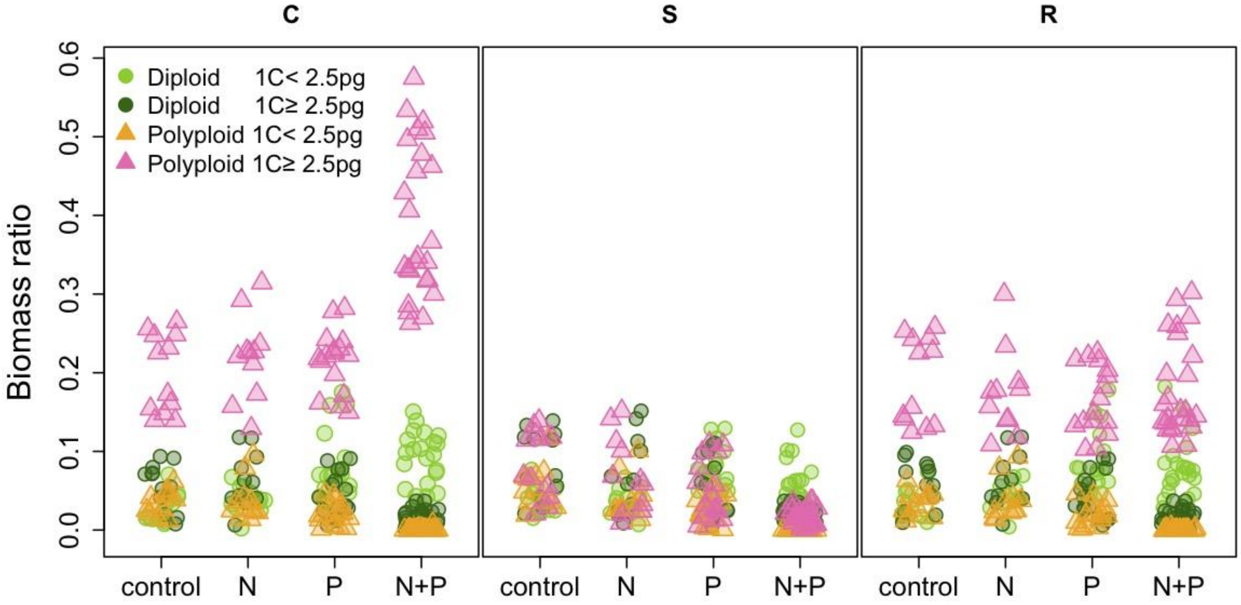

Fig. S10 Large GS ≥ 2.5 pg. Species above-ground biomass weighted by C-S-R strategy (C, competitor; S, stress-tolerant; R, ruderal). Each subplot is represented by four points corresponding to the four genomic parameter groups: diploids 1C-value < 2.5pg; diploids 1C-value ≥ 2.5pg; polyploids 1C-value < 2.5pg; and polyploids 1C-value ≥ 2.5pg. Polyploids with GS > 2.5pg were absent from 18 out of 24 N + P subplots (biomass ratio = 0). See also Tables S7 and S8.

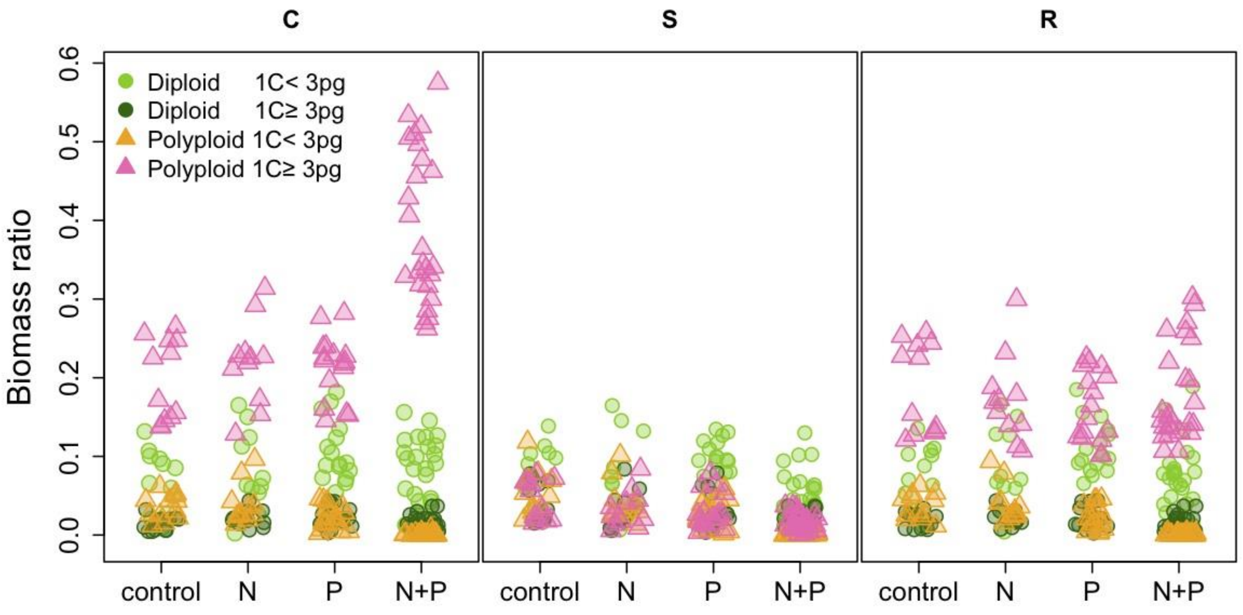

Fig. S11 Large GS ≥ 3 pg. Species biomass weighted by C-S-R strategy (C, competitor; S, stress-tolerant; R, ruderal), with a 3 pg threshold grouping taxa with large GS. Polyploids with GS ≥ 3pg were absent from nine out of 24 N + P subplots (biomass ratio = 0). See also Tables S9 and S10.

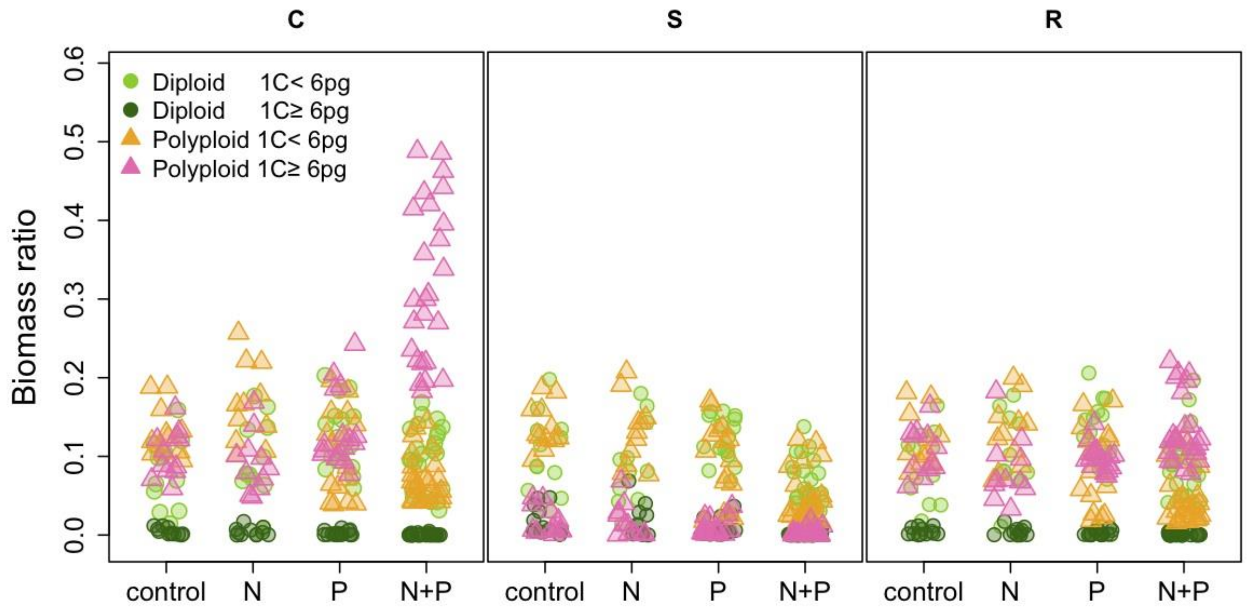

Fig. S12 Large GS threshold ≥ 6pg: species biomass weighted by C-S-R strategy (C, competitor; S, stress-tolerant; R, ruderal), where the large GS threshold is set at 6 pg: diploids 1C-value < 6pg; diploids 1C-value ≥ 6p; polyploids 1C-value < 6pg; and polyploids 1C-value ≥ 6pg. Diploids with GS ≥ 6pg were absent from seven N + P subplots. (biomass ratio = 0). See also Tables S11 and S12.

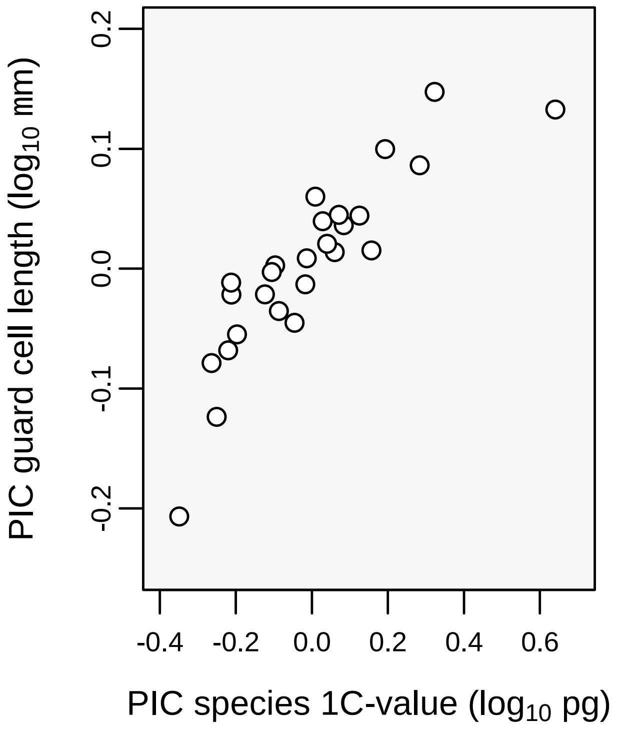

Fig. S13 Phylogeny-independent contrasts (PIC) on mean guard cell length and 1C-value in 27 taxa collected from the Park Grass Experiment plots. $R^2 = 0.761$, $F(1, 25) = 79.702$, $P < 0.00001$. Leaf imprints were made with clear varnish on fresh mature leaves and mounted on microscope slides. The length of 30 guard cells from leaves of two to 11 plants of each species were measured using OpenLab software and mean length estimated. Cell length and GS were $\log_{10}$ transformed and the association between PICs of guard cell sizes and PICs of 1C-values were tested in a linear regression through the origin with 10,000 permutations (functions *pic* and *lmorigin* from the *ape* package, Paradis *et al*. 2004).

Table S1 Full list of species occurring in the 64 subplots analysed at Park Grass together with family and the accession numbers of the sequences obtained from NCBI's GenBank to estimate a phylogenetic tree

ID	Species	Family	matK	rbcL	trnF-trnL	trnT-trnL	atpF-atpH
AM	*Achillea millefolium*	Asteraceae	HM850607.1	JX848399.1	AY603266.1	-	FJ395299.1
AU	*Agrimonia eupatorium*	Rosaceae	HM850683.1	JN891277.1	-	GQ384718.1	FJ395318.1
AC	*Agrostis capillaris*	Poaceae	JN895337.1	JN891522.1	EU119354.1	AY450936.1	FJ395329.1
AR	*Ajuga reptans*	Lamiaceae	AY840130.1	U32163.1	HQ911712.1	-	-
AP	*Alopecurus pratensis*	Poaceae	HM850564.1	HM849759.1	EU434101.1	-	EU434165.1
AO	*Anthoxanthum odoratum*	Poaceae	HM850562.1	HM849780.1	EF137590.1	-	FJ395289.1
AS	*Anthriscus sylvestris*	Apiaceae	U58547.1	JN893702.1	-	-	FJ395283.1
AE	*Arrhenatherum elatius*	Poaceae	EU434292.1	AY395529.1	-	DQ336866.1	FJ766100.1
BP	*Bellis perennis*	Asteraceae	HM850613.1	AY395530.1	JN315894.1	-	-
BM	*Briza media*	Poaceae	JN894143.1	AJ746285.1	DQ631446.1	DQ631512.1	-
BH	*Bromus hordeaceus*	Poaceae	HM850582.1	HM849826.1	EU036174.1	EU036148.1	GQ247913.1
CY	*Carex caryophyllea*	Cyperaceae	JN895022.1	JN892138.1	EU288430.1	-	-
CX	*Carex flacca*	Cyperaceae	JN895262.1	JN891463.1	DQ998968.1	-	-
CN	*Centaurea nigra*	Asteraceae	JN895499.1	JN893384.1	-	-	FJ395314.1
CF	*Cerastium fontanum*	Caryophyllaceae	HM850786.1	HM849881.1	AY521370.1	-	FJ395279.1
CM	*Conopodium majus*	Apiaceae	JN895810.1	JN893624.1	-	-	-
CC	*Crepis capillaris*	Asteraceae	JN895402.1	JN892652.1	-	-	-
CR	*Cynosurus cristatus*	Poaceae	HM850529.1	EF125151.1	EF137599.1	-	-
DG	*Dactylis glomerata*	Poaceae	HM850569.1	AY395535.1	AF533028.1	DQ631481.1	FJ395298.1
DC	*Deschampsia cespitosa*	Poaceae	JN894900.1	JX848495.1	AY237914.1	DQ631507.1	FJ395310.1
FP	*Festuca pratensis*	Poaceae	HM850535.1	JN891048.1	-	GU726883.1	-
FR	*Festuca rubra*	Poaceae	HQ593297.1	JN891471.1	-	DQ336857.1	FJ395301.1
FM	*Fritillaria meleagris*	Liliaceae	AY624445.1	AY395537.1	-	-	-
GV	*Galium verum*	Rubiaceae	JN893877.1	JN892891.1	-	-	HQ594712.1
HP	*Helictotrichon pubescens*	Poaceae	JN895846.1	JN891447.1	-	DQ631526.1	-
HS	*Heracleum sphondylium*	Apiaceae	JN894476.1	JN893491.1	-	AM998530.1	FJ395304.1
HI	*Hieracium pilosella*	Asteraceae	HE970711.1	JN891685.1	DQ460865.1	-	HQ594728.1
HL	*Holcus lanatus*	Poaceae	JN894527.1	JN892327.1	EF137606.1	DQ631503.1	FJ395300.1
HR	*Hypochaeris radicata*	Asteraceae	HM850666.1	HM850069.1	AF528380.1	-	FJ395296.1
KA	*Knautia arvensis*	Dipsacaceae	JN895237.1	JN892433.1	FJ640666.1	-	-
LP	*Lathyrus pratensis*	Fabaceae	JX505811.1	JN891335.1	JX505683.1	-	-
LA	*Leontodon autumnalis*	Asteraceae	JN895402.1	JN892652.1	AF528391.1	-	-
LH	*Leontodon hispidus*	Asteraceae	JN894007.1	JN890753.1	JQ041846.1	-	-
LM	*Lolium perenne*	Poaceae	HM850533.1	JN893059.1	EU119376.1	DQ367404.1	FJ766122.1
LO	*Lotus corniculatus*	Fabaceae	HM049505.1	JN892127.1	-	-	HQ594766.1
LC	*Luzula campestris*	Juncaceae	JN895446.1	HM850146.1	AY437943.1	-	FJ395316.1
OR	*Ononis repens*	Fabaceae	JN895637.1	JN890867.1	-	-	-
PS	*Pimpinella saxifraga*	Apiaceae	FR865050.1	JN892078.1	-	-	-

PL	*Plantago lanceolata*	Plantaginaceae	HE966968.1	JN893615.1	EU036272.1	-	GQ248030.1
PP	*Poa pratensis*	Poaceae	JN966444.1	JN965752.1	-	JF904790.1	FJ395325.1
PT	*Poa trivialis*	Poaceae	HM850517.1	JN893080.1	AY327795.1	-	FJ395264.1
PX	*Potentilla sterilis*	Rosaceae	JN895651.1	JN893010.1	FN561732.1	-	-
PV	*Primula veris*	Primulaceae	JN896058.1	AF394982.1	JQ927136.1	-	-
PZ	*Prunella vulgaris*	Lamiaceae	HM850805.1	AY395556.1	AY506619.1	-	-
QR	*Quercus robur*	Fagaceae	JN895518.1	FN675735.1	HM770040.1	-	-
RA	*Ranunculus acris*	Ranunculaceae	JN894744.1	JN965795.1	-	-	FJ395343.1
RU	*Ranunculus auricomus*	Ranunculaceae	JN894692.1	JN893758.1	-	-	-
RB	*Ranunculus bulbosus*	Ranunculaceae	HM851057.1	JN892326.1	FJ490812.1	-	FJ395281.1
RF	*Ranunculus ficaria*	Ranunculaceae	AY954232.1	EU053919.1	-	-	-
RC	*Rumex acetosa*	Polygonaceae	JN895619.1	JN893396.1	AJ583853.1	-	FJ395278.1
SM	*Sanguisorba minor*	Rosaceae	HM850691.1	JN892329.1	EU873351.1	-	-
SO	*Stachys officinalis*	Lamiaceae	JN896053.1	HE963693.1	FJ854224.1	-	-
SG	*Stellaria graminea*	Caryophyllaceae	JN895064.1	JN892194.1	AY521345.1	-	-
TO	*Taraxacum officinale*	Asteraceae	FJ395377.1	JX520956.1	EF015611.1	-	FJ395276.1
TG	*Tragopogon pratensis*	Asteraceae	JN893953.1	JN890681.1	JQ041858.1	JQ041828.1	FJ395355.1
TP	*Trifolium pratense*	Fabaceae	JN895372.1	JN893083.1	JQ041859.1	-	FJ395288.1
TR	*Trifolium repens*	Fabaceae	HE967014.1	JN892960.1	AB546814.1	-	FJ395344.1
TF	*Trisetum flavescens*	Poaceae	JN895340.1	JN893258.1	DQ336850.1	DQ336877.1	-
VC	*Veronica chamaedrys*	Plantaginaceae	JN894843.1	JN891876.1	AF486377.1	-	-
VR	*Viola riviniana*	Violaceae	JN894328.1	JN893557.1	-	-	-

Table S2 Full list of taxa occurring at Park Grass with GS (1C-values), chromosome number (2n), ploidy level (x), and C-S-R type, organized according to genomic grouping: diploids with 1C-value < 5pg, diploids with 1C-value ≥ 5pg, polyploids with 1C-value < 5pg, and polyploids with 1C-value ≥ 5pg. Where GS estimates were obtained for the present work, the SD is given together with the mean coefficient of variation (CoV, %) for the flow histogram peaks of both the target taxon and the internal standard. The number of plants we sampled to estimate GS are listed under the column heading (*n*). Other GS estimates were taken from Bennett & Leitch (2012) and are listed in the "Ref" column; see below this table for the authors. Where our CoV was high (e.g. >4), and if available, we used a previously published C-value that was closest to ours, and give our 1C-value estimate in the SD column (along with the SD). Overall, our dataset comprises: 29 diploid species with small GS (1C < 5 pg) (three monocot and nine eudicot families); 5 diploid species with big GS (1C ≥ 5 pg) (two monocot and one eudicot family); 17 polyploid species with small GS (two monocot and eight eudicot families); and nine polyploid species with big GS (one monocot and four eudicot families). Chrom num, chromosome number. na, not applicable.

ID	Species	Family	1C-value (pg)	1C-value SD	Mean CoV of target taxon	Mean CoV of internal standard	*n*	Chrom num (2n)	Ploidy level (x)	CSR type	*Ref
**Diploid 1C < 5 pg**											
AS	*Anthriscus sylvestris*	Apiaceae	2.25	2.18 ±0.04	5.27 ±1.11	2.32 ±0.24	3	16	2	C/CR	2
B	*Briza media*	Poaceae	3.35	0.07	2.29 ±0.97	2.43 ±0.86	19	na	2	S/CSR	-
BP	*Bellis perennis*	Asteraceae	1.15	-	-	-	-	18	2	R/CSR	13
CC	*Crepis capillaris*	Asteraceae	2.10	-	-	-	-	6	2	R/SR	7
C	*Conopodium majus*	Apiaceae	0.83	0.02	4.15 ±0.5	2.74 ±0.44	6	22	2	SR	-
CR	*Cynosurus cristatus*	Poaceae	3.05	-	-	-	-	14	2	R/CSR	18
CY	*Carex caryophyllea*	Cyperac.	0.78	-	-	-	-	66	2	S/CSR	11
FP	*Festuca pratensis*	Poaceae	2.23	-	-	-	-	14	2	CSR	16
HI	*Hieracium pilosella*	Asteraceae	3.53	0.06	2.8 ±0.31	2.59 ±0.67	8	18	2	S/CSR	-
HL	*Holcus lanatus*	Poaceae	1.89	0.12	2.48 ±0.67	2.16 ±0.54	50	14	2	CSR	-
HR	*Hypochaeris radicata*	Asteraceae	1.34	-	-	-	-	8	2	CSR	6
HS	*Heracleum sphondylium*	Apiaceae	2.19	2.56 ±0.13	3.42 ±0.80	2.78 ±0.18	2	22	2	C/CSR	17
LA	*Leontodon autumnalis*	Asteraceae	1.16	-	-	-	-	12	2	R/CSR	17
LC	*Luzula campestris*	Juncaceae	0.43	0.02	3.89 ±0.77	2.62 ±0.76	2	12	2	S/CSR	-
LH	*Leontodon hispidus*	Asteraceae	2.50	0.07	4.51 ±0.09	2.17 ±0.1	2	14	2	CSR	5
LM	*Lolium perenne*	Poaceae	3.06	0.03	3.23 ±0.3	3.12 ±0.17	3	14	2	CR/CSR	-
PL	*Plantago lanceolata*	Plantagin.	1.20	-	4.09 ±NA	3.51 ±NA	1	12	2	CSR	11
PS	*Pimpinella saxifraga*	Apiaceae	3.89	0.04	4.63 ±2.22	3.13 ±0.76	3	20	2	SR/CSR	-
PT	*Poa trivialis*	Poaceae	1.85	0.03	4.17 ±1.15	2.72 ±0.94	13	14	2	R/CSR	-
PV	*Primula veris*	Primula.	0.49	-	-	-	-	22	2	S/CSR	17
PZ	*Prunella vulgaris*	Lamiaceae	0.65	-	-	-	-	28	2	CSR	23
QR	*Quercus robur*	Fagaceae	0.93	-	-	-	-	24	2	SC	8
RA	*Ranunculus acris*	Ranuncul.	4.74	0.19	3.8 ±1.01	2.92 ±0.7	21	14	2	CSR	-
RC	*Rumex acetosa*	Polygon.	3.55	0.07	2.28 ±0.56	2.39 ±0.41	7	14	2	CSR	-
SG	*Stellaria graminea*	Caryophyll.	1.17	NA	3.13	2.61	1	26	2	CSR	-

SO	*Stachys officinalis*	Lamiaceae	4.53	4.91 ±NA	4.19 ±NA	3.3 ±NA	1	16	2	S/CSR	2
TG	*Tragopogon pratensis*	Asteraceae	2.77	2.53 ±NA	4.93 ±NA	2.03 ±NA	1	12	2	CR/CSR	14
TO	*Taraxacum officinale*	Asteraceae	1.53	0.28	3.77 ±1.14	2.89 ±1	18	16	2	R/CSR	-
TP	*Trifolium pratense*	Fabaceae	0.53	0.51 ±NA	3.17 ±NA	2.11 ±NA	1	14	2	CSR	3
**Diploid 1C ≥ 5 pg**											
DC	*Deschampsia cespitosa*	Poaceae	5.22	-	-	-	-	26	2	SC/CSR	12
F	*Fritillaria meleagris*	Liliaceae	47.30	-	-	-	-	24	2	SR	10
HP	*Helictotrichon pubescens*	Poaceae	6.28	-	-	-	-	14	2	S/CSR	15
RB	*Ranunculus bulbosus*	Ranuncul.	5.63	-	-	-	-	16	2	SR	19
RF	*Ranunculus ficaria*	Ranuncul.	9.33	-	-	-	-	16	2	SR	20
**Polyploid < 5 pg**											
AC	*Agrostis capillaris*	Poaceae	3.53	-	-	-	15	28	4	CSR	11
AR	*Ajuga reptans*	Lamiaceae	1.19	0.04	3.09 ±0.75	2.89 ±0.61	9	32	poly	R/CSR	-
AU	*Agrimonia eupatorium*	Rosaceae	3.98	0.01	3.33 ±0.34	3.04 ±0.42	3	28	poly	CSR	-
CF	*Cerastium fontanum*	Caryophyll.	2.93	-	-	-	-	144	8, 16	R/CSR	4
CN	*Centaurea nigra*	Asteraceae	2.25	0.04	3.11 ±0.57	2.56 ±0.42	4	44	poly	CSR	-
CX	*Carex flacca*	Cyperaceae	0.30	-	-	-	-	76	4	S/SC	9
DG	*Dactylis glomerata*	Poaceae	4.15	0.11	4.38 ±1.28	3.62 ±0.74	9	28	4	C/CSR	-
GV	*Galium verum*	Rubiaceae	1.89	2.10	5.22	2.79	5	44	4	SC/CSR	23
LO	*Lotus corniculatus*	Fabaceae	1.27	0.08	3.78 ±0.38	3.37 ±1.13	24	24	4	S/CSR	-
OR	*Ononis repens*	Fabaceae	1.41	0.01	4.79 ±0.79	3.16 ±0.52	6	various	poly	SC/CSR	-
PP	*Poa pratensis*	Poaceae	4.90	1.08	3.33 ±1.12	3.46 ±0.9	31	various	poly	CSR	-
PX	*Potentilla sterilis*	Rosaceae	0.97	-	-	-	1	28	poly	SR/CSR	-
S	*Sanguisorba minor*	Rosaceae	0.55	0.62 ±NA	4.85 ±NA	2.93 ±NA	1	28	4	S/CSR	9
TF	*Trisetum flavescens*	Poaceae	2.55	-	-	-	-	various	poly	CSR	2
TR	*Trifolium repens*	Fabaceae	1.29	0.22	2.41 ±0.31	2.06 ±0.35	8	32	4	CR/CSR	-
VC	*Veronica chamaedrys*	Plantagin.	1.49	-	-	-	-	32	4	CSR	1
VR	*Viola riviniana*	Violaceae	1.35	-	-	-	-	40	4	S/CSR	9
**Polyploid ≥ 5 pg**											
AE	*Arrhenatherum elatius*	Poaceae	7.99	0.22	2.82 ±0.87	3.09 ±0.46	31	28	4	C/CSR	-
A	*Achillea millefolium*	Asteraceae	8.37	0.07	3.09 ±0.66	2.24 ±0.62	49	54	6	CSR	-
AO	*Anthoxanthum odoratum*	Poaceae	6.31	0.21	2.04 ±0.43	2.33 ±0.45	67	20	4	SR/CSR	-
AP	*Alopecurus pratensis*	Poaceae	6.80	-	-	-	-	28	4	C/CSR	13
BH	*Bromus hordeaceus*	Poaceae	11.06	0.06	2.99 ±0.78	2.97 ±0.69	5	28	4	R/CR	-
FR	*Festuca rubra*	Poaceae	6.13	0.14	2.02 ±0.52	2.45 ±0.47	45	42	6	CSR	-
KA	*Knautia arvensis*	Dipsaca.	7.01	7.09 ±0.23	3.31 ±0.45	2.96 ±0.16	10	40	4	CSR	22
LP	*Lathyrus pratensis*	Fabaceae	11.46	0.43	3.34 ±0.99	3.7 ±0.82	-	na	poly	CSR	-
RU	*Ranunculus auricomus*	Ranuncul.	9.00	-	-	-	-	32	4	SR	21

*Reference sources for the C-values used in the present work that were taken from the Plant DNA C-values database (Bennett & Leitch 2012). The database source references were: (1) Albach & Greilhuber (2004) (reference 478); (2) S. R. Band (pers. comm.) (reference 154); (3) Barow & Meister (2003) (reference 492); (4) Bennett et al. (*1982*) (reference 105); (5) M. A. Callimassia & M. D. Bennett (pers. comm.) (reference 269-H); (6) Cerbah *et al.* (1999) (reference 666); (7) Evans *et al*. (1972) (reference 7); (8) Favre & Brown (1996) (reference 366); (9) Grime *et al*. (1985) (reference 133); (10) Leitch *et al.* (2007) (reference 565); (11) Mowforth (1986) (reference 158); (12) Murray *et al*. (2005) (reference 528); (13) Olszewska &

Osiecka (1982) (reference 155); (13) Olszewska & Osiecka (1983) (reference 156); (14) Pires *et al.* (2004) (reference 510); (15) Roser (1995) (reference 283); (16) A. G. Seal (pers. comm.) (reference 72); (17) Siljak-Yakovlev *et al*. (2010) (reference 634); (18) Šmarda *et al.* (2008) (reference 555); (19) Smith & Bennett (1975) (reference 45); (20) Smith & Bennett (1975) (reference 45); (21) Smith & Bennett (1975) (reference 45); (22) Temsch & Greilhuber (2010) (reference 652); (23) Temsch *et al.* (2010) (reference 627).

Table S3 Measures of arithmetic mean and SD under four different nutrient treatments: (control i.e. no fertilizer); N (N without P); P (P without N); N + P (both N and P applied) on: mean biomass (aboveground dry weight), biomass-weighted mean 1C-value, biomass-weighted mean $1C_{pgls}$ (phylogenetic least squares); presence-absence (i.e. unweighted by biomass) mean $1C_{pgls}$. *n*, number of subplots in each treatment. $1C_{pgls}$ means were $\log_{10}$ back-transformed to facilitate interpretation. $1C_{pgls}$ presence-absence are unweighted by biomass. Mean $1C_{pgls}$ were estimated as in Šmarda *et al*. (2013), using the *gls* function in the *nlme* package (Pinheiro *et al.*, 2013) and the *ape* package (Paradis *et al.*, 2004). *Pgls* accommodates models with phylogenetic correlation structures and we used it to obtain for each subplot a phylogeny-adjusted biomass-weighted mean GS (= 'biomass-weighted mean $1C_{pgls}$-value') which takes into account each taxon's contribution to total biomass, and a presence-absence mean which is unweighted by species biomass. We calculated $1C_{pgls}$ by using the R formula y~1 to estimate subplot means where y = the 1C-value of each taxon; a within group structure was specified by Brownian motion phylogenetic covariance, and biomass fraction, or presence-absence, described the within-group heteroscedasticity. A phylogenetic tree of 60 species was used to estimate both types of mean $1C_{pgls}$-value for each subplot (see Methods S1 for phylogenetic tree file). A biomass value of 0.000001 was attributed to absent species.

Treatment	*n*	Total biomass (g)	Spp no.	Biomass-weighted mean 1C (pg)	Biomass-weighted mean $1C_{pgls}$ (pg)	Presence-absence mean $1C_{pgls}$ (pg)
Control	12	31.71 ± 4.27	39 ± 4.2	3.99 ± 0.37	3.19 ± 0.81	2.12 ± 0.14
N	11	34.47 ± 3.9	31 ± 5.6	3.87 ± 0.51	3.24 ± 0.73	2.13 ± 0.54
P	16	44.49 ± 11.75	32 ± 4.2	4.17 ± 0.44	2.66 ± 0.5	2.24 ± 0.36
N + P	25	58.36 ± 11.64	20 ± 5.5	5.4 ± 0.52	5.07 ± 1.28	2.81 ± 0.42

Table S4 Means and ratios with SD per nutrient treatment for:(a) total subplot biomass; (b) total number of species per subplot, and (c) total C-weighted; (d) total S-weighted; (e) total R-weighted biomass for the four genomic groups of taxa: diploid taxa with small GS; diploid taxa with big GS, polyploid taxa with small GS; and polyploid taxa with big GS. 1C = 1C-value (pg). The number of subplots per treatment is as follows: control = 12, N = 11, P = 16, N + P = 25.

**(a)**	**Mean total biomass (g)**				**Mean ratio total biomass**			
Treatment	Diploid 1C< 5pg	Diploid 1C≥ 5pg	Polyploid 1C< 5pg	Polyploid 1C≥ 5pg	Diploid 1C< 5pg	Diploid 1C≥ 5pg	Polyploid 1C< 5pg	Polyploid 1C≥ 5pg
Control	8.78 ±4.95	1.15 ±1.1	12.06 ±2.41	9.72 ±2.02	0.267 ±0.14	0.033 ±0.03	0.385 ±0.09	0.314 ±0.09
N	9.91 ±4.74	0.89 ±1.06	14.93 ±6.01	8.74 ±3.8	0.291 ±0.15	0.027 ±0.03	0.425 ±0.13	0.257 ±0.12
P	17.22 ±8.62	0.56 ±0.59	11.8 ±6.89	14.9 ±6.19	0.376 ±0.11	0.015 ±0.02	0.282 ±0.16	0.327 ±0.06
N+P	14.84 ±6.91	0.2 ±0.35	8.96 ±4.06	34.35 ±10.31	0.249 ±0.1	0.004 ±0.01	0.163 ±0.09	0.584 ±0.11

**(b)**	**Mean total number of species**				**Mean ratio total species number**			
Treatment	Diploid 1C< 5pg	Diploid 1C≥ 5pg	Polyploid 1C< 5pg	Polyploid 1C≥ 5pg	Diploid 1C< 5pg	Diploid 1C≥ 5pg	Polyploid 1C< 5pg	Polyploid 1C≥ 5pg
Control	19 ±3	2 ±0	11 ±2	7 ±1	0.49 ±0.04	0.052 ±0.01	0.29 ±0.03	0.168 ±0.02
N	15 ±4	2 ±0	8 ±2	6 ±1	0.485 ±0.05	0.056 ±0.02	0.268 ±0.02	0.191 ±0.05
P	15 ±2	1 ±1	9 ±2	7 ±0	0.476 ±0.03	0.043 ±0.01	0.266 ±0.04	0.215 ±0.02
N+P	10 ±3	1 ±1	4 ±1	5 ±2	0.48 ±0.05	0.037 ±0.03	0.218 ±0.05	0.265 ±0.04

**(c)**	**C-weighted mean total biomass (g)**				**C-weighted mean ratio**			
Treatment	Diploid 1C< 5pg	Diploid 1C≥ 5pg	Polyploid 1C< 5pg	Polyploid 1C≥ 5pg	Diploid 1C< 5pg	Diploid 1C≥ 5pg	Polyploid 1C< 5pg	Polyploid 1C≥ 5pg
Control	2.64 ±1.57	0.16 ±0.17	4.069 ±0.85	3.185 ±0.67	0.08 ±0.04	0.005 ±0	0.13 ±0.03	0.103 ±0.03
N	3.336 ±1.59	0.152 ±0.17	5.836 ±2.39	3.138 ±1.23	0.098 ±0.05	0.005 ±0.01	0.165 ±0.05	0.092 ±0.04
P	5.819 ±3.37	0.092 ±0.1	4.361 ±2.45	6.294 ±3.90	0.125 ±0.04	0.002 ±0	0.102 ±0.05	0.132 ±0.05
N+P	5.918 ±3.04	0.034 ±0.06	4.134 ±1.83	19.548 ±9.10	0.097 ±0.04	0.001 ±0	0.072 ±0.03	0.32 ±0.1

**(d)**	**S-weighted mean total biomass (g)**				**S-weighted mean ratio**			
Treatment	Diploid 1C< 5pg	Diploid 1C≥ 5pg	Polyploid 1C< 5pg	Polyploid 1C≥ 5pg	Diploid 1C< 5pg	Diploid 1C≥ 5pg	Polyploid 1C< 5pg	Polyploid 1C≥ 5pg
Control	3.316 ±1.77	0.733 ±0.72	4.312 ±0.86	3.268 ±0.68	0.101 ±0.05	0.021 ±0.02	0.137 ±0.03	0.106 ±0.03
N	3.068 ±1.63	0.589 ±0.71	4.574 ±1.86	2.793 ±1.29	0.091 ±0.05	0.018 ±0.02	0.131 ±0.04	0.082 ±0.04
P	5.422 ±2.13	0.373 ±0.39	3.801 ±2.23	4.291 ±1.27	0.121 ±0.03	0.01 ±0.01	0.092 ±0.05	0.097 ±0.02
N + P	3.718 ±1.72	0.136 ±0.23	2.416 ±1.41	7.342 ±1.59	0.065 ±0.03	0.003 ±0	0.046 ±0.03	0.131 ±0.04

**(e)**	**R-weighted mean total biomass (g)**				**R-weighted mean ratio**			
Treatment	Diploid 1C< 5pg	Diploid 1C≥ 5pg	Polyploid 1C< 5pg	Polyploid 1C≥ 5pg	Diploid 1C< 5pg	Diploid 1C≥ 5pg	Polyploid 1C< 5pg	Polyploid 1C≥ 5pg
Control	2.822 ±1.63	0.252 ±0.22	3.68 ±0.86	3.268 ±0.68	0.086 ±0.04	0.007 ±0.01	0.118 ±0.03	0.106 ±0.03

N	3.502 ±1.6	0.151 ±0.18	4.518 ±1.83	2.812 ±1.31	0.102 ±0.05	0.005 ±0.01	0.129 ±0.04	0.083 ±0.04
P	5.979 ±3.16	0.099 ±0.1	3.638 ±2.27	4.316 ±1.3	0.13 ±0.04	0.003 ±0	0.087 ±0.05	0.098 ±0.02
N+P	5.208 ±2.67	0.034 ±0.06	2.412 ±1.41	7.464 ±1.65	0.087 ±0.04	0.001 ±0	0.046 ±0.03	0.133 ±0.04

Table S5 Treatment contrasts and ANOVA output testing the effects and interactions of N, P, GS (small vs large, where large GS ≥ 5 pg), ploidy (diploid vs polyploid), on square-root transformed subplot dependent variables: (a) total biomass; (b) total species numbers; (d) C-weighted biomass; (e) S-weighted biomass; and (f) R-weighted biomass testing for significance in treatment, GS, and ploidy. Part (c) shows multivariate ANOVA output where each C-S-R-weighted biomass are the response variables (i.e. *n* = 3). Baseline levels in the contrasts are: without N, without P, diploid, and small GS for N, P, GS and ploidy effects respectively (e.g. there is a 0.2 g increase in biomass with the addition of N, relative to subplots without N).

(a) Biomass

	Estimate	Std. error	t-value	Pr(>|t|)	ANOVA	df	F-value	*P*-value
(Intercept)	2.833	0.209	13.564	<0.0001	(Intercept)	1, 180	4171.593	<0.0001
N	0.202	0.302	0.667	0.5072	N	1, 60	6.101	0.0164
P	1.212	0.276	4.385	<0.0001	P	1, 60	24.497	<0.0001
GS	-1.884	0.295	-6.378	<0.0001	GS	1, 180	111.167	<0.0001
ploidy	0.624	0.295	2.111	0.0361	ploidy	1, 180	361.882	<0.0001
N : P	-0.506	0.381	-1.33	0.1885	N : P	1, 60	1.084	0.3019
N : GS	-0.368	0.427	-0.861	0.3904	N : GS	1, 180	11.836	0.0007
P : GS	-1.497	0.391	-3.83	0.0002	P : GS	1, 180	7.649	0.0063
N : ploidy	0.138	0.427	0.323	0.7468	N : ploidy	1, 180	18.713	<0.0001
P : ploidy	-1.364	0.391	-3.491	0.0006	P : ploidy	1, 180	4.092	0.0446
GS : ploidy	1.529	0.418	3.659	0.0003	GS : ploidy	1, 180	476.754	<0.0001
N : P : GS	0.311	0.538	0.577	0.5648	N : P : GS	1, 180	18.067	<0.0001
N : P : ploidy	-0.209	0.538	-0.388	0.6983	N : P : ploidy	1, 180	8.328	0.0044
N : GS : ploidy	-0.177	0.604	-0.292	0.7703	N : GS : ploidy	1, 180	27.93	<0.0001
P : GS : ploidy	2.34	0.553	4.234	<0.0001	P : GS : ploidy	1, 180	95.669	<0.0001
N : P : GS : ploidy	2.615	0.761	3.435	0.0007	N : P : GS : ploidy	1, 180	11.798	0.0007

(b) Species number

	Estimate	Std. error	t-value	Pr(>|t|)	ANOVA	df	F-value	*P*-value
(Intercept)	4.356	0.098	44.548	<0.0001	(Intercept)	1, 180	6513.509	<0.0001
N	-0.478	0.141	-3.379	0.0013	N	1, 60	77.91	<0.0001
P	-0.458	0.129	-3.545	0.0008	P	1, 60	44.253	<0.0001
GS	-2.941	0.138	-21.272	<0.0001	GS	1, 180	1719.748	<0.0001
ploidy	-1.01	0.138	-7.301	<0.0001	ploidy	1, 180	28.194	<0.0001
N : P	-0.326	0.178	-1.829	0.0723	N : P	1, 60	5.2	0.0262
N : GS	0.365	0.2	1.824	0.0242	N : GS	1, 180	46.525	<0.0001
P : GS	0.2	0.183	1.091	0.1758	P : GS	1, 180	33.378	<0.0001
N : ploidy	0.007	0.2	0.035	0.9649	N : ploidy	1, 180	0.258	0.6118
P : ploidy	0.027	0.183	0.149	0.853	P : ploidy	1, 180	7.81	0.0058
GS : ploidy	2.142	0.196	10.951	<0.0001	GS : ploidy	1, 180	1227.501	<0.0001
N : P : GS	0.02	0.252	0.08	0.9207	N : P : GS	1, 180	0.763	0.3836
N : P : ploidy	-0.057	0.252	-0.226	0.7783	N : P : ploidy	1, 180	0.111	0.739
N : GS : ploidy	-0.054	0.283	-0.19	0.8133	N : GS : ploidy	1, 180	0.873	0.3515

P : GS : ploidy	0.294	0.259	1.138	0.1581	P : GS : ploidy	1, 180	8.032	0.0051
N : P : GS : ploidy	0.21	0.356	0.588	0.4649	N : P : GS : ploidy	1, 180	0.536	0.4649

(c) C-S-R

	Estimate	Std. error	t-value	Pr(>|t|)	ANOVA	df	F-value	*P*-value
(Intercept)	4.897	0.351	13.941	<0.0001	(Intercept)	1, 180	4311.457	<0.0001
N	0.35	0.508	0.69	0.493	N	1, 60	4.514	0.0377
P	2.103	0.465	4.525	<0.0001	P	1, 60	22.648	<0.0001
GS	-3.34	0.497	-6.724	<0.0001	GS	1, 180	127.655	<0.0001
ploidy	1.083	0.497	2.18	0.0305	ploidy	1, 180	371.2	<0.0001
N : P	-0.923	0.64	-1.442	0.1544	N : P	1, 60	0.613	0.4367
N : GS	-0.624	0.718	-0.868	0.3863	N : GS	1, 180	11.467	0.0009
P : GS	-2.574	0.657	-3.917	0.0001	P : GS	1, 180	7.245	0.0078
N : ploidy	0.23	0.718	0.32	0.7493	N : ploidy	1, 180	16.275	0.0001
P : ploidy	-2.374	0.657	-3.613	0.0004	P : ploidy	1, 180	2.899	0.0903
GS : ploidy	2.732	0.703	3.888	0.0001	GS : ploidy	1, 180	495.404	<0.0001
N : P : GS	0.604	0.905	0.667	0.5057	N : P : GS	1, 180	18.09	<0.0001
N : P : ploidy	-0.375	0.905	-0.414	0.6793	N : P : ploidy	1, 180	7.423	0.0071
N : GS : ploidy	-0.316	1.016	-0.311	0.7562	N : GS : ploidy	1, 180	25.867	<0.0001
P : GS : ploidy	3.999	0.929	4.303	<0.0001	P : GS : ploidy	1, 180	95.052	<0.0001
N : P : GS : ploidy	4.238	1.28	3.31	0.0011	N : P : GS : ploidy	1, 180	10.957	0.0011

(d) C

	Estimate	Std. error	t-value	Pr(>|t|)	ANOVA	df	F-value	*P*-value
(Intercept)	1.543	0.158	9.785	<0.0001	(Intercept)	1, 180	2306.12	<0.0001
N	0.214	0.228	0.94	0.3508	N	1, 60	24.796	<0.0001
P	0.789	0.209	3.781	0.0004	P	1, 60	38.771	<0.0001
GS	-1.197	0.223	-5.37	<0.0001	GS	1, 180	58.754	<0.0001
ploidy	0.464	0.223	2.082	0.0313	ploidy	1, 180	411.583	<0.0001
N : P	-0.204	0.287	-0.711	0.4801	N : P	1, 60	3.489	0.0667
N : GS	-0.231	0.322	-0.716	0.4561	N : GS	1, 180	14.344	0.0002
P : GS	-0.867	0.295	-2.937	0.0025	P : GS	1, 180	13.775	0.0003
N : ploidy	0.152	0.322	0.47	0.6247	N : ploidy	1, 180	28.87	<0.0001
P : ploidy	-0.778	0.295	-2.636	0.0066	P : ploidy	1, 180	11.163	0.001
GS : ploidy	0.965	0.315	3.061	0.0017	GS : ploidy	1, 180	425.739	<0.0001
N : P : GS	0.076	0.406	0.187	0.8453	N : P : GS	1, 180	18.824	<0.0001
N : P : ploidy	-0.189	0.406	-0.465	0.6282	N : P : ploidy	1, 180	11.399	0.0009
N : GS : ploidy	-0.169	0.456	-0.371	0.699	N : GS : ploidy	1, 180	35.335	<0.0001
P : GS : ploidy	1.494	0.417	3.582	0.0003	P : GS : ploidy	1, 180	94.449	<0.0001
N : P : GS : ploidy	2.239	0.575	3.895	0.0001	N : P : GS : ploidy	1, 180	16.502	0.0001

(e) S

	Estimate	Std. error	t-value	Pr(>|t|)	ANOVA	df	F-value	*P*-value

(Intercept)	1.753	0.113	15.552	<0.0001	(Intercept)	1, 180	4286.365	<0.0001
N	-0.07	0.163	-0.428	0.67	N	1, 60	3.18	0.0796
P	0.537	0.149	3.602	0.0006	P	1, 60	2.458	0.1222
GS	-0.998	0.159	-6.259	<0.0001	GS	1, 180	118.499	<0.0001
ploidy	0.314	0.159	1.967	0.0507	ploidy	1, 180	261.774	<0.0001
N : P	-0.34	0.205	-1.656	0.1029	N : P	1, 60	0.065	0.7998
N : GS	-0.054	0.231	-0.235	0.8146	N : GS	1, 180	11.517	0.0008
P : GS	-0.754	0.211	-3.577	0.0004	P : GS	1, 180	4.303	0.0395
N : ploidy	0.104	0.231	0.451	0.6526	N : ploidy	1, 180	11.367	0.0009
P : ploidy	-0.735	0.211	-3.486	0.0006	P : ploidy	1, 180	1.121	0.2912
GS : ploidy	0.73	0.225	3.236	0.0014	GS : ploidy	1, 180	349.74	<0.0001
N : P : GS	0.172	0.291	0.593	0.5539	N : P : GS	1, 180	11.28	0.001
N : P : ploidy	-0.062	0.291	-0.213	0.8315	N : P : ploidy	1, 180	4.922	0.0278
N : GS : ploidy	-0.145	0.326	-0.445	0.657	N : GS : ploidy	1, 180	13.477	0.0003
P : GS : ploidy	1.207	0.298	4.047	0.0001	P : GS : ploidy	1, 180	73.004	<0.0001
N : P : GS : ploidy	1.035	0.411	2.52	0.0126	N : P : GS : ploidy	1, 180	6.349	0.0126

(f) R

	Estimate	Std. error	t-value	Pr(>|t|)	ANOVA	df	F-value	*P*-value
(Intercept)	1.601	0.115	13.887	<0.0001	(Intercept)	1, 180	3952.054	<0.0001
N	0.206	0.167	1.234	0.222	N	1, 60	0.395	0.5321
P	0.777	0.153	5.094	<0.0001	P	1, 60	12.097	0.0009
GS	-1.145	0.163	-7.022	<0.0001	GS	1, 180	188.478	<0.0001
ploidy	0.305	0.163	1.873	0.0627	ploidy	1, 180	264.955	<0.0001
N : P	-0.379	0.21	-1.804	0.0763	N : P	1, 60	0.045	0.8329
N : GS	-0.339	0.236	-1.437	0.1525	N : GS	1, 180	4.131	0.0436
P : GS	-0.953	0.216	-4.418	<0.0001	P : GS	1, 180	1.706	0.1932
N : ploidy	-0.026	0.236	-0.108	0.9138	N : ploidy	1, 180	3.799	0.0529
P : ploidy	-0.861	0.216	-3.994	0.0001	P : ploidy	1, 180	0.052	0.8194
GS : ploidy	1.037	0.231	4.496	<0.0001	GS : ploidy	1, 180	504.959	<0.0001
N : P : GS	0.355	0.297	1.196	0.2333	N : P : GS	1, 180	15.884	0.0001
N : P : ploidy	-0.124	0.297	-0.417	0.677	N : P : ploidy	1, 180	2.904	0.0901
N : GS : ploidy	-0.002	0.333	-0.005	0.9962	N : GS : ploidy	1, 180	16.904	0.0001
P : GS : ploidy	1.298	0.305	4.254	<0.0001	P : GS : ploidy	1, 180	74.063	<0.0001
N : P : GS : ploidy	0.964	0.42	2.294	0.0229	N : P : GS : ploidy	1, 180	5.264	0.0229

Table S6 ANOVA output testing the effect of N and P treatment on *pgls* mean GS of each subplot : a) phylogenetic least squares biomass–weighted mean GS (= 'biomass-weighted mean $1C_{pgls}$-value') which takes into account each taxon's contribution to total biomass; and (b) phylogeny-adjusted 'presence-absence mean $1C_{pgls}$-value', where biomass was not taken into account. The intercepts below show the estimated *pgls* mean subplot GS without N or P treatment (control plots), and the estimated coefficients in the second column show the effects of N application (i.e. without P), the effects of P application (i.e. without N), and the effects when both are applied on a subplot. The reference level is no application of N or P. Significant parameters are in bold (*P*-value < 0.05).

**(a) Biomass-weighted mean $1C_{pgls}$**								
	Estimate	Std. error	t-value	Pr(>|t|)	ANOVA:	df	F-value	*P*-value
intercept	3.192	0.296	10.785	<0.00001	intercept	1, 60	885.1916	<0.0001
N	0.052	0.428	0.121	0.90373	**N**	**1, 60**	**36.0665**	**<0.0001**
P	-0.504	0.397	-1.269	0.20940	**P**	**1, 60**	**6.7855**	**0.0116**
**N : P**	**2.250**	**0.542**	**4.151**	**0.00011**	**N : P**	**1, 60**	**17.2289**	**0.0001**

**(b) Presence-absence mean $1C_{pgls}$**								
	Estimate	Std. error	t-value	Pr(>|t|)	ANOVA:	df	F-value	*P*-value
intercept	2.136	0.114	18.743	<0.00001	intercept	1, 60	2436.64	<0.0001
N	0.001	0.165	0.003	0.99750	**N**	**1, 60**	**20.5973**	**<0.0001**
P	0.068	0.153	0.444	0.65850	**P**	**1, 60**	**15.2558**	**0.0002**
**N : P**	**0.630**	**0.209**	**3.021**	**0.00370**	**N : P**	**1, 60**	**9.1268**	**0.0037**

Table S7 Different 1C-value thresholds for small vs large GS were investigated. This table shows means, ratios, and standard deviations where large GS ≥ 2.5 pg between the four genomic groups for: (a) total subplot biomass; (b) total number of species per subplot, and (c) total C-weighted; (d) total S-weighted; (e) total R-weighted biomass. As above, the four genomic groups of taxa are: diploid taxa with small GS; diploid taxa with big GS; polyploid taxa with small GS; and polyploid taxa with big GS. 1C= 1C-value (pg). The number of subplots per treatment is as follows: control = 12, N = 11, P = 16, N + P = 25. See also Figs S7 and S10.

**(a)**	**Mean total biomass (g)**				**Mean ratio total biomass**			
Treatment	Diploid 1C< 2.5pg	Diploid 1C≥ 2.5pg	Polyploid 1C< 2.5pg	Polyploid 1C≥ 2.5pg	Diploid 1C< 2.5pg	Diploid 1C≥ 2.5pg	Polyploid 1C< 2.5pg	Polyploid 1C≥ 2.5pg
Control	3.293 ±2.09	6.63 ±3.77	3.959 ±1.96	17.825 ±3.44	0.099 ±0.06	0.201 ±0.1	0.122 ±0.05	0.578 ±0.16
N	4.1 ±1.66	6.697 ±3.61	4.052 ±3.15	19.62 ±6.16	0.12 ±0.05	0.198 ±0.12	0.115 ±0.08	0.567 ±0.16
P	12.206 ±8.7	5.579 ±3.16	2.706 ±2.17	23.995 ±6.54	0.251 ±0.11	0.14 ±0.09	0.064 ±0.05	0.545 ±0.1
N+P	12.218 ±6.8	2.83 ±1.6	0.068 ±0.18	43.246 ±9.63	0.202 ±0.11	0.051 ±0.03	0.001 ±0	0.746 ±0.11

**(b)**	**Mean total number of species**				**Mean ratio species number**			
Treatment	Diploid 1C< 2.5pg	Diploid 1C≥ 2.5pg	Polyploid 1C< 2.5pg	Polyploid 1C≥ 2.5pg	Diploid 1C< 2.5pg	Diploid 1C≥ 2.5pg	Polyploid 1C< 2.5pg	Polyploid 1C≥ 2.5pg
Control	12 ±2	9 ±1	39 ±4	6 ±1	0.307 ±0.03	0.234 ±0.02	0.167 ±0.02	0.291 ±0.03
N	9 ±2	8 ±2	31 ±5	3 ±1	0.291 ±0.04	0.251 ±0.02	0.107 ±0.03	0.352 ±0.07
P	10 ±1	7 ±2	32 ±4	4 ±2	0.304 ±0.04	0.215 ±0.03	0.113 ±0.04	0.368 ±0.03
N+P	7 ±2	4 ±2	20 ±5	0 ±1	0.333 ±0.06	0.184 ±0.06	0.018 ±0.04	0.465 ±0.06

**(c)**	**C-weighted mean total biomass (g)**				**C-weighted mean biomass ratio**			
Treatment	Diploid 1C< 2.5pg	Diploid 1C≥ 2.5pg	Polyploid 1C< 2.5pg	Polyploid 1C≥ 2.5pg	Diploid 1C< 2.5pg	Diploid 1C≥ 2.5pg	Polyploid 1C< 2.5pg	Polyploid 1C≥ 2.5pg
Control	0.957 ±0.67	1.843 ±1.07	1.097 ±0.53	6.157 ±1.05	0.029 ±0.02	0.056 ±0.03	0.034 ±0.02	0.199 ±0.05
N	1.331 ±0.57	2.157 ±1.1	1.328 ±1.06	7.646 ±2.44	0.039 ±0.02	0.063 ±0.04	0.038 ±0.03	0.219 ±0.05
P	4.135 ±3.36	1.776 ±0.95	0.846 ±0.71	9.809 ±3.88	0.083 ±0.05	0.044 ±0.03	0.02 ±0.01	0.215 ±0.04
N+P	5.018 ±2.95	0.934 ±0.53	0.025 ±0.07	23.657 ±9.63	0.081 ±0.04	0.017 ±0.01	0.001 ±0	0.391 ±0.1

**(d)**	**S-weighted mean total biomass (g)**				**S-weighted mean biomass ratio**			
Treatment	Diploid 1C< 2.5pg	Diploid 1C≥ 2.5pg	Polyploid 1C< 2.5pg	Polyploid 1C≥ 2.5pg	Diploid 1C< 2.5pg	Diploid 1C≥ 2.5pg	Polyploid 1C< 2.5pg	Polyploid 1C≥ 2.5pg
Control	1.224 ±0.69	2.825 ±1.62	1.764 ±1	5.816 ±1.21	0.037 ±0.02	0.085 ±0.04	0.054 ±0.03	0.189 ±0.06
N	1.287 ±0.53	2.37 ±1.54	1.407 ±1.07	5.96 ±1.93	0.038 ±0.02	0.071 ±0.05	0.04 ±0.03	0.173 ±0.05
P	3.785 ±2.2	2.009 ±1.26	1.025 ±0.76	7.066 ±1.8	0.08 ±0.03	0.051 ±0.04	0.025 ±0.02	0.164 ±0.04
N + P	2.893 ±1.79	0.961 ±0.55	0.025 ±0.07	9.733 ±1.83	0.05 ±0.03	0.017 ±0.01	0.001 ±0	0.176 ±0.06

**(e)**	**R-weighted mean total biomass (g)**				**R-weighted mean biomass ratio**			

Treatment	Diploid 1C< 2.5pg	Diploid 1C≥ 2.5pg	Polyploid 1C< 2.5pg	Polyploid 1C≥ 2.5pg	Diploid 1C< 2.5pg	Diploid 1C≥ 2.5pg	Polyploid 1C< 2.5pg	Polyploid 1C≥ 2.5pg
Control	1.112 ±0.73	1.962 ±1.1	1.098 ±0.54	5.85 ±1.19	0.033 ±0.02	0.06 ±0.03	0.034 ±0.02	0.19 ±0.06
N	1.482 ±0.62	2.171 ±1.1	1.317 ±1.03	6.013 ±1.97	0.043 ±0.02	0.064 ±0.04	0.037 ±0.03	0.174 ±0.05
P	4.286 ±3.17	1.793 ±0.97	0.835 ±0.72	7.119 ±1.78	0.088 ±0.04	0.045 ±0.03	0.02 ±0.01	0.166 ±0.04
N+P	4.307 ±2.6	0.935 ±0.53	0.018 ±0.04	9.857 ±1.85	0.071 ±0.04	0.017 ±0.01	0 ±0	0.178 ±0.06

Table S8 Large GS ≥ 2.5 pg. Treatment contrasts and ANOVA output testing the effects and interactions of N, P, GS (small vs large, where large GS ≥ 2.5 pg), and ploidy (diploid vs polyploid), on subplot: (a) total biomass; (b) total species numbers; (d) C-weighted biomass; (e) S-weighted biomass; and (f) R-weighted biomass testing for significance in treatment, GS, and ploidy. Part (c) shows multivariate ANOVA output where each C-S-R-weighted biomass are the response variables (i.e. *n* = 3). Baseline levels in the contrasts are: without N, without P, diploid, and small GS for N, P, GS and ploidy effects respectively. Dependent variables (i.e. total biomass, species numbers, C, S, R-weighted biomass) were square-root transformed.

(a) Biomass

	Estimate	Std. error	t-value	Pr(>|t|)	ANOVA	df	F-value	*P*-value
(Intercept)	1.727	0.209	8.283	<0.0001	(Intercept)	1, 180	3903.145	<0.0001
N	0.237	0.302	0.787	0.4345	N	1, 60	0.039	0.8436
P	1.588	0.276	5.757	<0.0001	P	1, 60	10.182	0.0023
GS	0.724	0.295	2.456	0.015	GS	1, 180	371.789	<0.0001
ploidy	0.202	0.295	0.685	0.4941	ploidy	1, 180	74.589	<0.0001
N : P	-0.232	0.380	-0.612	0.543	N : P	1, 60	1.089	0.3009
N : GS	-0.210	0.426	-0.492	0.6232	N : GS	1, 180	18.915	<0.0001
P : GS	-1.780	0.390	-4.562	<0.0001	P : GS	1, 180	2.214	0.1385
N : ploidy	-0.258	0.426	-0.605	0.5459	N : ploidy	1, 180	1.756	0.1868
P : ploidy	-2.006	0.390	-5.143	<0.0001	P : ploidy	1, 180	2.570	0.1106
GS : ploidy	1.549	0.417	3.715	0.0003	GS : ploidy	1, 180	738.763	<0.0001
N : P : GS	-0.459	0.537	-0.854	0.3944	N : P : GS	1, 180	10.145	0.0017
N : P : ploidy	-1.142	0.537	-2.125	0.0349	N : P : ploidy	1, 180	1.924	0.1671
N : GS : ploidy	0.409	0.603	0.679	0.4981	N : GS : ploidy	1, 180	71.494	<0.0001
P : GS : ploidy	2.849	0.552	5.163	<0.0001	P : GS : ploidy	1, 180	147.459	<0.0001
N : P : GS : ploidy	3.339	0.760	4.392	<0.0001	N : P : GS : ploidy	1, 180	19.294	<0.0001

(b) Species number

	Estimate	Std. error	t-value	Pr(>|t|)	ANOVA	df	F-value	*P*-value
(Intercept)	3.450	0.107	32.112	<0.0001	(Intercept)	1, 180	4776.047	<0.0001
N	-0.449	0.155	-2.889	0.0054	N	1, 60	93.639	<0.0001
P	-0.343	0.142	-2.413	0.0189	P	1, 60	52.885	<0.0001
GS	-0.442	0.110	-4.006	0.0001	GS	1, 180	385.281	<0.0001
ploidy	-0.906	0.110	-8.222	<0.0001	ploidy	1, 180	126.444	<0.0001
N : P	-0.093	0.196	-0.475	0.6363	N : P	1, 60	7.586	0.0078
N : GS	0.221	0.159	1.390	0.1664	N : GS	1, 180	55.622	<0.0001

	Estimate	Std. error	t-value	Pr(>|t|)	ANOVA	df	F-value	*P*-value
P : GS	-0.047	0.146	-0.322	0.7481	P : GS	1, 180	27.087	<0.0001
N : ploidy	-0.279	0.159	-1.748	0.0821	N : ploidy	1, 180	12.714	0.0005
P : ploidy	-0.319	0.146	-2.187	0.03	P : ploidy	1, 180	0.898	0.3446
GS : ploidy	1.248	0.156	8.003	<0.0001	GS : ploidy	1, 180	1188.565	<0.0001
N : P : GS	-0.394	0.201	-1.963	0.0512	N : P : GS	1, 180	0.140	0.7089
N : P : ploidy	-0.706	0.201	-3.514	0.0006	N : P : ploidy	1, 180	3.313	0.0704
N : GS : ploidy	0.414	0.225	1.837	0.0678	N : GS : ploidy	1, 180	69.089	<0.0001
P : GS : ploidy	0.776	0.206	3.763	0.0002	P : GS : ploidy	1, 180	77.362	<0.0001
N : P : GS : ploidy	0.895	0.284	3.150	0.0019	N : P : GS : ploidy	1, 180	9.921	0.0019

(c) C-S-R

	Estimate	Std. error	t-value	Pr(>|t|)	ANOVA	df	F-value	*P*-value
(Intercept)	2.984	0.353	8.446	<0.0001	(Intercept)	1, 180	4000.850	<0.0001
N	0.412	0.511	0.806	0.4236	N	1, 60	0.027	0.8708
P	2.752	0.467	5.888	<0.0001	P	1, 60	8.830	0.0043
GS	1.238	0.500	2.479	0.0141	GS	1, 180	374.461	<0.0001
ploidy	0.328	0.500	0.656	0.5127	ploidy	1, 180	71.691	<0.0001
N : P	-0.464	0.644	-0.721	0.4735	N : P	1, 60	1.801	0.1846
N : GS	-0.356	0.722	-0.493	0.6225	N : GS	1, 180	17.357	<0.0001
P : GS	-3.064	0.661	-4.636	<0.0001	P : GS	1, 180	1.678	0.1969
N : ploidy	-0.420	0.722	-0.581	0.5621	N : ploidy	1, 180	1.220	0.2708
P : ploidy	-3.454	0.661	-5.227	<0.0001	P : ploidy	1, 180	3.556	0.061
GS : ploidy	2.729	0.707	3.862	0.0002	GS : ploidy	1, 180	744.348	<0.0001
N : P : GS	-0.739	0.910	-0.812	0.4181	N : P : GS	1, 180	9.677	0.0022
N : P : ploidy	-1.938	0.910	-2.129	0.0347	N : P : ploidy	1, 180	1.558	0.2136
N : GS : ploidy	0.658	1.022	0.644	0.5206	N : GS : ploidy	1, 180	67.367	<0.0001
P : GS : ploidy	4.850	0.935	5.189	<0.0001	P : GS : ploidy	1, 180	144.930	<0.0001
N : P : GS : ploidy	5.483	1.288	4.258	<0.0001	N : P : GS : ploidy	1, 180	18.135	<0.0001

(d) C

	Estimate	Std. error	t-value	Pr(>|t|)	ANOVA	df	F-value	*P*-value
(Intercept)	0.919	0.156	5.881	<0.0001	(Intercept)	1, 180	2216.498	<0.0001
N	0.193	0.226	0.855	0.396	N	1, 60	9.008	0.0039
P	0.981	0.207	4.745	<0.0001	P	1, 60	21.906	<0.0001
GS	0.367	0.213	1.719	0.0874	GS	1, 180	351.150	<0.0001
ploidy	0.097	0.213	0.453	0.6514	ploidy	1, 180	107.827	<0.0001
N : P	0.014	0.285	0.048	0.9617	N : P	1, 60	0.030	0.8641

					ANOVA	df	F-value	P-value
N : GS	-0.069	0.308	-0.225	0.8224	N : GS	1, 180	25.348	<0.0001
P : GS	-0.987	0.282	-3.498	0.0006	P : GS	1, 180	6.941	0.0092
N : ploidy	-0.120	0.308	-0.389	0.6981	N : ploidy	1, 180	6.730	0.0103
P : ploidy	-1.153	0.282	-4.087	0.0001	P : ploidy	1, 180	0.002	0.9682
GS : ploidy	1.090	0.302	3.614	0.0004	GS : ploidy	1, 180	697.041	<0.0001
N : P : GS	-0.501	0.389	-1.288	0.1993	N : P : GS	1, 180	10.430	0.0015
N : P : ploidy	-0.861	0.389	-2.216	0.028	N : P : ploidy	1, 180	3.678	0.0567
N : GS : ploidy	0.258	0.436	0.591	0.5556	N : GS : ploidy	1, 180	83.273	<0.0001
P : GS : ploidy	1.762	0.399	4.415	<0.0001	P : GS : ploidy	1, 180	138.086	<0.0001
N : P : GS : ploidy	2.777	0.550	5.052	<0.0001	N : P : GS : ploidy	1, 180	25.519	<0.0001

(e) S

	Estimate	Std. error	t-value	Pr(>|t|)	ANOVA	df	F-value	*P*-value
(Intercept)	1.065	0.113	9.406	<0.0001	(Intercept)	1, 180	3958.037	<0.0001
N	0.039	0.164	0.239	0.812	N	1, 60	15.067	0.0003
P	0.811	0.150	5.417	<0.0001	P	1, 60	0.002	0.9659
GS	0.539	0.160	3.364	0.0009	GS	1, 180	338.458	<0.0001
ploidy	0.215	0.160	1.345	0.1802	ploidy	1, 180	49.929	<0.0001
N : P	-0.301	0.206	-1.460	0.1494	N : P	1, 60	3.961	0.0511
N : GS	-0.192	0.232	-0.828	0.4089	N : GS	1, 180	14.325	0.0002
P : GS	-1.069	0.212	-5.048	<0.0001	P : GS	1, 180	0.539	0.4637
N : ploidy	-0.191	0.232	-0.827	0.4093	N : ploidy	1, 180	0.311	0.5778
P : ploidy	-1.162	0.212	-5.485	<0.0001	P : ploidy	1, 180	5.911	0.016
GS : ploidy	0.580	0.226	2.562	0.0112	GS : ploidy	1, 180	542.580	<0.0001
N : P : GS	0.036	0.292	0.124	0.9015	N : P : GS	1, 180	8.425	0.0042
N : P : ploidy	0.407	0.292	1.395	0.1648	N : P : ploidy	1, 180	0.570	0.4512
N : GS : ploidy	0.359	0.327	1.095	0.2749	N : GS : ploidy	1, 180	46.252	<0.0001
P : GS : ploidy	1.659	0.300	5.537	<0.0001	P : GS : ploidy	1, 180	119.450	<0.0001
N : P : GS : ploidy	1.126	0.413	2.727	0.007	N : P : GS : ploidy	1, 180	7.439	0.007

(f) R

	Estimate	Std. error	t-value	Pr(>|t|)	ANOVA	df	F-value	*P*-value
(Intercept)	1.000	0.119	8.422	<0.0001	(Intercept)	1, 180	3613.775	<0.0001
N	0.179	0.172	1.044	0.3005	N	1, 60	1.269	0.2643
P	0.959	0.157	6.110	<0.0001	P	1, 60	4.121	0.0468
GS	0.333	0.168	1.985	0.0486	GS	1, 180	263.553	<0.0001
ploidy	0.016	0.168	0.094	0.9253	ploidy	1, 180	27.726	<0.0001

N : P	-0.177	0.216	-0.818	0.4169	N : P	1, 60	5.499	0.0223
N : GS	-0.095	0.243	-0.393	0.6951	N : GS	1, 180	5.722	0.0178
P : GS	-1.007	0.222	-4.537	<0.0001	P : GS	1, 180	0.037	0.8472
N : ploidy	-0.108	0.243	-0.446	0.6561	N : ploidy	1, 180	0.292	0.5894
P : ploidy	-1.139	0.222	-5.131	<0.0001	P : ploidy	1, 180	11.182	0.001
GS : ploidy	1.058	0.237	4.457	<0.0001	GS : ploidy	1, 180	646.927	<0.0001
N : P : GS	-0.274	0.306	-0.897	0.3712	N : P : GS	1, 180	5.693	0.0181
N : P : ploidy	-0.670	0.306	-2.189	0.0299	N : P : ploidy	1, 180	0.311	0.5776
N : GS : ploidy	0.041	0.343	0.121	0.9041	N : GS : ploidy	1, 180	40.272	<0.0001
P : GS : ploidy	1.429	0.314	4.551	<0.0001	P : GS : ploidy	1, 180	109.699	<0.0001
N : P : GS : ploidy	1.581	0.433	3.654	0.0003	N : P : GS : ploidy	1, 180	13.352	0.0003

Table S9 Large GS ≥ 3 pg. Means, ratios, and SD per nutrient treatment for: (a) total subplot biomass; (b) total number of species per subplot, and (c) total C-weighted; (d) total S-weighted; (e) total R-weighted biomass for the four genomic groupings. See also Figs S8 and S11.

**(a)**	**Mean total biomass (g)**				**Mean ratio total biomass**			
Treatment	Diploid 1C< 3pg	Diploid 1C≥ 3pg	Polyploid 1C< 3pg	Polyploid 1C≥ 3pg	Diploid 1C< 3pg	Diploid 1C≥ 3pg	Polyploid 1C< 3pg	Polyploid 1C≥ 3pg
Control	7.357 ±4.2	2.566 ±1.63	4.153 ±2.13	17.631 ±3.55	0.223 ±0.11	0.077 ±0.04	0.127 ±0.06	0.572 ±0.17
N	8.205 ±4.79	2.593 ±1.33	4.199 ±3.09	19.473 ±6.2	0.243 ±0.15	0.075 ±0.04	0.119 ±0.08	0.562 ±0.16
P	14.651 ±7.76	3.134 ±1.78	3.025 ±2.2	23.677 ±6.74	0.317 ±0.09	0.074 ±0.05	0.072 ±0.05	0.537 ±0.11
N+P	12.452 ±6.96	2.596 ±1.53	0.13 ±0.22	43.184 ±9.66	0.206 ±0.11	0.047 ±0.03	0.003 ±0	0.745 ±0.11

**(b)**	**Mean total number of species**				**Mean ratio species number**			
Treatment	Diploid 1C< 3pg	Diploid 1C≥ 3pg	Polyploid 1C< 3pg	Polyploid 1C≥ 3pg	Diploid 1C< 3pg	Diploid 1C≥ 3pg	Polyploid 1C< 3pg	Polyploid 1C≥ 3pg
Control	14 ±2	8 ±1	8 ±1	10 ±1	0.345 ±0.03	0.196 ±0.03	0.208 ±0.02	0.251 ±0.03
N	11 ±3	6 ±1	5 ±2	9 ±1	0.34 ±0.04	0.201 ±0.03	0.159 ±0.04	0.3 ±0.07
P	11 ±1	6 ±1	6 ±2	10 ±1	0.348 ±0.04	0.171 ±0.03	0.168 ±0.05	0.313 ±0.03
N+P	7 ±2	3 ±1	6 ±2	8 ±2	0.358 ±0.06	0.159 ±0.05	0.058 ±0.06	0.425 ±0.06

**(c)**	**C-weighted mean total biomass (g)**				**C-weighted mean biomass ratio**			
Treatment	Diploid 1C< 3pg	Diploid 1C≥ 3pg	Polyploid 1C< 3pg	Polyploid 1C≥ 3pg	Diploid 1C< 3pg	Diploid 1C≥ 3pg	Polyploid 1C< 3pg	Polyploid 1C≥ 3pg
Control	2.323 ±1.39	0.477 ±0.33	1.15 ±0.57	6.104 ±1.08	0.07 ±0.04	0.014 ±0.01	0.036 ±0.02	0.197 ±0.05
N	2.737 ±1.61	0.751 ±0.45	1.366 ±1.04	7.609 ±2.46	0.081 ±0.05	0.021 ±0.01	0.039 ±0.03	0.218 ±0.05
P	4.975 ±3.08	0.936 ±0.5	0.943 ±0.71	9.712 ±3.93	0.106 ±0.04	0.022 ±0.01	0.022 ±0.01	0.212 ±0.04
N+P	5.115 ±3.01	0.836 ±0.5	0.045 ±0.08	23.638 ±9.64	0.083 ±0.04	0.015 ±0.01	0.001 ±0	0.391 ±0.1

**(d)**	**S-weighted mean total biomass (g)**				**S-weighted mean biomass ratio**			
Treatment	Diploid 1C< 3pg	Diploid 1C≥ 3pg	Polyploid 1C< 3pg	Polyploid 1C≥ 3pg	Diploid 1C< 3pg	Diploid 1C≥ 3pg	Polyploid 1C< 3pg	Polyploid 1C≥ 3pg
Control	2.556 ±1.38	1.493 ±0.95	1.817 ±1.04	5.763 ±1.24	0.078 ±0.04	0.045 ±0.03	0.055 ±0.03	0.187 ±0.06
N	2.58 ±1.59	1.076 ±0.77	1.444 ±1.06	5.922 ±1.95	0.077 ±0.05	0.032 ±0.02	0.041 ±0.03	0.172 ±0.05
P	4.549 ±1.83	1.245 ±0.79	1.122 ±0.78	6.969 ±1.87	0.101 ±0.02	0.03 ±0.02	0.027 ±0.02	0.162 ±0.05
N + P	2.932 ±1.83	0.922 ±0.54	0.045 ±0.08	9.713 ±1.84	0.05 ±0.03	0.017 ±0.01	0.001 ±0	0.176 ±0.06

**(e)**	**R-weighted mean total biomass (g)**				**R-weighted mean biomass ratio**			
Treatment	Diploid 1C< 3pg	Diploid 1C≥ 3pg	Polyploid 1C< 3pg	Polyploid 1C≥ 3pg	Diploid 1C< 3pg	Diploid 1C≥ 3pg	Polyploid 1C< 3pg	Polyploid 1C≥ 3pg
Control	2.477 ±1.44	0.596 ±0.36	1.185 ±0.6	5.763 ±1.24	0.075 ±0.04	0.018 ±0.01	0.036 ±0.02	0.187 ±0.06
N	2.887 ±1.62	0.765 ±0.44	1.388 ±0.99	5.941 ±1.98	0.085 ±0.05	0.022 ±0.01	0.039 ±0.02	0.172 ±0.05
P	5.126 ±2.88	0.953 ±0.51	0.959 ±0.72	6.994 ±1.86	0.11 ±0.03	0.022 ±0.01	0.023 ±0.02	0.163 ±0.04
N+P	4.404 ±2.68	0.838 ±0.5	0.04 ±0.06	9.835 ±1.86	0.073 ±0.04	0.015 ±0.01	0.001 ±0	0.178 ±0.06

Table S10 Large GS ≥ 3 pg. Treatment contrasts and ANOVA output testing the effects and interactions of N, P, GS (small vs large, where large GS ≥ 3 pg), ploidy (diploid vs polyploid), on square-root transformed subplot dependent variables of: (a) total biomass; (b) total species numbers; (d) C-weighted biomass; (e) S-weighted biomass; and (f) R-weighted biomass testing for significance in treatment, GS, and ploidy. Part (c) shows multivariate ANOVA output where each C-S-R-weighted biomass are the response variables (i.e. n = 3). Baseline levels in the contrasts are: without N, without P, diploid, and small GS for N, P, GS and ploidy effects respectively (e.g. there is a 0.2 g increase in biomass with the addition of N, relative to subplots without N).

(a) Biomass

	Estimate	Std. error	t-value	Pr(>|t|)	ANOVA	df	F-value	*P*-value
(Intercept)	2.582	0.204	12.662	<0.0001	(Intercept)	1, 180	4059.450	<0.0001
N	0.130	0.295	0.442	0.6597	N	1, 60	0.180	0.6729
P	1.140	0.270	4.227	0.0001	P	1, 60	12.523	0.0008
GS	-1.066	0.288	-3.695	0.0003	GS	1, 180	195.728	<0.0001
ploidy	-0.610	0.288	-2.115	0.0358	ploidy	1, 180	96.050	<0.0001
N : P	-0.504	0.372	-1.355	0.1804	N : P	1, 60	0.556	0.4588
N : GS	-0.091	0.417	-0.217	0.8283	N : GS	1, 180	43.379	<0.0001
P : GS	-0.967	0.381	-2.535	0.0121	P : GS	1, 180	21.261	<0.0001
N : ploidy	-0.148	0.417	-0.354	0.7237	N : ploidy	1, 180	1.717	0.1917
P : ploidy	-1.487	0.381	-3.899	0.0001	P : ploidy	1, 180	2.370	0.1254
GS : ploidy	3.273	0.408	8.025	<0.0001	GS : ploidy	1, 180	1044.294	<0.0001
N : P : GS	0.302	0.525	0.575	0.5662	N : P : GS	1, 180	18.678	<0.0001
N : P : ploidy	-0.876	0.525	-1.667	0.0973	N : P : ploidy	1, 180	1.327	0.2508
N : GS : ploidy	0.293	0.590	0.497	0.62	N : GS : ploidy	1, 180	43.361	<0.0001
P : GS : ploidy	1.952	0.539	3.619	0.0004	P : GS : ploidy	1, 180	80.368	<0.0001
N : P : GS : ploidy	2.608	0.743	3.509	0.0006	N : P : GS : ploidy	1, 180	12.313	0.0006

(b) Species number

	Estimate	Std. error	t-value	Pr(>|t|)	ANOVA	df	F-value	*P*-value
(Intercept)	3.661	0.113	32.256	<0.0001	(Intercept)	1, 180	4875.466	<0.0001
N	-0.413	0.164	-2.518	0.0145	N	1, 60	78.190	<0.0001
P	-0.339	0.150	-2.261	0.0274	P	1, 60	43.865	<0.0001
GS	-0.913	0.124	-7.350	<0.0001	GS	1, 180	13.348	0.0003
ploidy	-0.825	0.124	-6.643	<0.0001	ploidy	1, 180	40.566	<0.0001
N : P	-0.244	0.207	-1.179	0.2432	N : P	1, 60	6.066	0.0167
N : GS	0.156	0.180	0.868	0.3863	N : GS	1, 180	53.523	<0.0001

P : GS	-0.069	0.164	-0.420	0.6747	P : GS	1, 180	25.397	<0.0001
N : ploidy	-0.205	0.180	-1.140	0.2557	N : ploidy	1, 180	3.810	0.0525
P : ploidy	-0.190	0.164	-1.158	0.2483	P : ploidy	1, 180	0.118	0.7319
GS : ploidy	1.184	0.176	6.739	<0.0001	GS : ploidy	1, 180	741.941	<0.0001
N : P : GS	-0.071	0.226	-0.312	0.7556	N : P : GS	1, 180	3.160	0.0772
N : P : ploidy	-0.553	0.226	-2.442	0.0156	N : P : ploidy	1, 180	1.525	0.2185
N : GS : ploidy	0.353	0.254	1.388	0.167	N : GS : ploidy	1, 180	36.376	<0.0001
P : GS : ploidy	0.644	0.232	2.770	0.0062	P : GS : ploidy	1, 180	40.567	<0.0001
N : P : GS : ploidy	0.710	0.320	2.218	0.0278	N : P : GS : ploidy	1, 180	4.921	0.0278

(c) C-S-R

	Estimate	Std. error	t-value	Pr(>|t|)	ANOVA	df	F-value	*P*-value
(Intercept)	4.469	0.345	12.972	<0.0001	(Intercept)	1, 180	4176.040	<0.0001
N	0.222	0.498	0.446	0.6572	N	1, 60	0.008	0.9299
P	1.971	0.456	4.326	0.0001	P	1, 60	11.461	0.0013
GS	-1.930	0.487	-3.961	0.0001	GS	1, 180	193.245	<0.0001
ploidy	-1.080	0.487	-2.217	0.0279	ploidy	1, 180	94.740	<0.0001
N : P	-0.930	0.628	-1.482	0.1437	N : P	1, 60	1.150	0.2879
N : GS	-0.104	0.705	-0.148	0.8825	N : GS	1, 180	42.347	<0.0001
P : GS	-1.592	0.645	-2.470	0.0145	P : GS	1, 180	20.809	<0.0001
N : ploidy	-0.227	0.705	-0.322	0.7479	N : ploidy	1, 180	1.106	0.2943
P : ploidy	-2.552	0.645	-3.959	0.0001	P : ploidy	1, 180	3.572	0.0604
GS : ploidy	5.778	0.689	8.385	<0.0001	GS : ploidy	1, 180	1066.735	<0.0001
N : P : GS	0.537	0.888	0.605	0.546	N : P : GS	1, 180	18.035	<0.0001
N : P : ploidy	-1.479	0.888	-1.666	0.0975	N : P : ploidy	1, 180	1.073	0.3018
N : GS : ploidy	0.413	0.996	0.415	0.6789	N : GS : ploidy	1, 180	39.251	<0.0001
P : GS : ploidy	3.234	0.912	3.547	0.0005	P : GS : ploidy	1, 180	76.339	<0.0001
N : P : GS : ploidy	4.258	1.256	3.391	0.0009	N : P : GS : ploidy	1, 180	11.501	0.0009

(d) C

	Estimate	Std. error	t-value	Pr(>|t|)	ANOVA	df	F-value	*P*-value
(Intercept)	1.442	0.154	9.357	<0.0001	(Intercept)	1, 180	2145.746	<0.0001
N	0.118	0.223	0.528	0.5991	N	1, 60	10.156	0.0023
P	0.706	0.204	3.463	0.001	P	1, 60	24.380	<0.0001
GS	-0.793	0.208	-3.815	0.0002	GS	1, 180	204.517	<0.0001
ploidy	-0.403	0.208	-1.940	0.0539	ploidy	1, 180	134.705	<0.0001
N : P	-0.138	0.281	-0.492	0.6247	N : P	1, 60	0.109	0.7421
N : GS	0.068	0.301	0.228	0.8202	N : GS	1, 180	49.820	<0.0001

P : GS	-0.427	0.275	-1.552	0.1223	P : GS	1, 180	29.332	<0.0001
N : ploidy	-0.045	0.301	-0.150	0.8807	N : ploidy	1, 180	6.401	0.0123
P : ploidy	-0.837	0.275	-3.044	0.0027	P : ploidy	1, 180	0.000	0.9862
GS : ploidy	2.217	0.294	7.538	<0.0001	GS : ploidy	1, 180	968.394	<0.0001
N : P : GS	-0.108	0.379	-0.286	0.7751	N : P : GS	1, 180	16.538	0.0001
N : P : ploidy	-0.709	0.379	-1.871	0.063	N : P : ploidy	1, 180	3.332	0.0696
N : GS : ploidy	0.124	0.425	0.293	0.7701	N : GS : ploidy	1, 180	56.565	<0.0001
P : GS : ploidy	1.155	0.389	2.968	0.0034	P : GS : ploidy	1, 180	81.635	<0.0001
N : P : GS : ploidy	2.396	0.536	4.471	<0.0001	N : P : GS : ploidy	1, 180	19.993	<0.0001

(e) S

	Estimate	Std. error	t-value	Pr(>|t|)	ANOVA	df	F-value	*P*-value
(Intercept)	1.532	0.111	13.846	<0.0001	(Intercept)	1, 180	4157.026	<0.0001
N	-0.011	0.160	-0.072	0.9432	N	1, 60	14.683	0.0003
P	0.565	0.146	3.863	0.0003	P	1, 60	0.028	0.8686
GS	-0.376	0.156	-2.401	0.0174	GS	1, 180	179.992	<0.0001
ploidy	-0.234	0.156	-1.498	0.1358	ploidy	1, 180	62.095	<0.0001
N : P	-0.462	0.202	-2.292	0.0254	N : P	1, 60	2.762	0.1017
N : GS	-0.167	0.226	-0.738	0.4612	N : GS	1, 180	34.986	<0.0001
P : GS	-0.665	0.207	-3.212	0.0016	P : GS	1, 180	13.983	0.0002
N : ploidy	-0.139	0.226	-0.613	0.5406	N : ploidy	1, 180	0.415	0.5203
P : ploidy	-0.875	0.207	-4.228	<0.0001	P : ploidy	1, 180	4.860	0.0288
GS : ploidy	1.466	0.221	6.623	<0.0001	GS : ploidy	1, 180	789.182	<0.0001
N : P : GS	0.493	0.285	1.728	0.0857	N : P : GS	1, 180	17.068	0.0001
N : P : ploidy	-0.244	0.285	-0.855	0.3934	N : P : ploidy	1, 180	0.228	0.6334
N : GS : ploidy	0.335	0.320	1.047	0.2964	N : GS : ploidy	1, 180	24.492	<0.0001
P : GS : ploidy	1.205	0.293	4.116	0.0001	P : GS : ploidy	1, 180	60.296	<0.0001
N : P : GS : ploidy	0.681	0.403	1.688	0.0932	N : P : GS : ploidy	1, 180	2.848	0.0932

(f) R

		S	t	P				
	stimate	td. error	-value	r(>|t|)	ANOVA	df	F-value	*P*-value
(Intercept)	1.495	0.116	12.884	<0.0001	(Intercept)	1, 180	3734.925	<0.0001
N	0.116	0.168	0.690	0.4925	N	1, 60	0.722	0.399
P	0.700	0.154	4.561	<0.0001	P	1, 60	6.297	0.0148
GS	-0.761	0.164	-4.637	<0.0001	GS	1, 180	107.337	<0.0001
ploidy	-0.442	0.164	-2.695	0.0077	ploidy	1, 180	44.613	<0.0001
N : P	-0.330	0.212	-1.561	0.1238	N : P	1, 60	4.380	0.0406

New Phytologist

N : GS	-0.006	0.237	-0.024	0.9811	N : GS	1, 180	22.444	<0.0001
P : GS	-0.500	0.217	-2.303	0.0224	P : GS	1, 180	9.709	0.0021
N : ploidy	-0.043	0.237	-0.181	0.8568	N : ploidy	1, 180	0.486	0.4868
P : ploidy	-0.839	0.217	-3.866	0.0002	P : ploidy	1, 180	12.160	0.0006
GS : ploidy	2.095	0.232	9.027	<0.0001	GS : ploidy	1, 180	945.730	<0.0001
N : P : GS	0.153	0.299	0.511	0.61	N : P : GS	1, 180	12.367	0.0006
N : P : ploidy	-0.526	0.299	-1.759	0.0803	N : P : ploidy	1, 180	0.094	0.7597
N : GS : ploidy	-0.046	0.336	-0.138	0.8902	N : GS : ploidy	1, 180	18.946	<0.0001
P : GS : ploidy	0.874	0.307	2.847	0.0049	P : GS : ploidy	1, 180	50.246	<0.0001
N : P : GS : ploidy	1.182	0.423	2.794	0.0058	N : P : GS : ploidy	1, 180	7.807	0.0058

Table S11 Large GS ≥ 6 pg. Means, ratios, and SD per nutrient treatment for: (a) total subplot biomass; (b) total number of species per subplot, and (c) total C-weighted; (d) total S-weighted; (e) total R-weighted biomass for the four genomic groupings. See also Figs S9 and S12.

**(a)**	**Mean total biomass (g)**				**Mean ratio total biomass**			
Treatment	Diploid 1C< 6pg	Diploid 1C≥ 6pg	Polyploid 1C< 6pg	Polyploid 1C≥ 6pg	Diploid 1C< 6pg	Diploid 1C≥ 6pg	Polyploid 1C< 6pg	Polyploid 1C≥ 6pg
Control	8.961 ±4.99	0.962 ±1.03	12.061 ±2.41	9.722 ±2.02	0.273 ±0.14	0.027 ±0.03	0.385 ±0.09	0.314 ±0.09
N	9.928 ±4.73	0.87 ±1.07	14.929 ±6.01	8.743 ±3.8	0.292 ±0.15	0.027 ±0.03	0.425 ±0.13	0.257 ±0.12
P	17.237 ±8.61	0.548 ±0.59	11.801 ±6.89	14.901 ±6.19	0.377 ±0.11	0.014 ±0.02	0.282 ±0.16	0.327 ±0.06
N+P	14.845 ±6.91	0.203 ±0.35	8.962 ±4.06	34.352 ±10.3	0.249 ±0.1	0.004 ±0.01	0.163 ±0.09	0.584 ±0.11

**(b)**	**Mean total number of species**				**Mean ratio species number**			
Treatment	Diploid 1C< 6pg	Diploid 1C≥ 6pg	Polyploid 1C< 6pg	Polyploid 1C≥ 6pg	Diploid 1C< 6pg	Diploid 1C≥ 6pg	Polyploid 1C< 6pg	Polyploid 1C≥ 6pg
Control	20 ±3	1 ±0	11 ±2	6 ±1	0.516 ±0.04	0.026 ±0	0.29 ±0.03	0.168 ±0.02
N	16 ±4	1 ±0	8 ±2	6 ±1	0.503 ±0.05	0.039 ±0.01	0.268 ±0.02	0.191 ±0.05
P	16 ±2	1 ±0	9 ±2	7 ±0	0.486 ±0.03	0.034 ±0.01	0.266 ±0.04	0.215 ±0.02
N+P	10 ±3	1 ±1	4 ±1	5 ±2	0.48 ±0.05	0.038 ±0.03	0.218 ±0.05	0.265 ±0.04

**(c)**	**C-weighted mean total biomass (g)**				**C-weighted mean biomass ratio**			
Treatment	Diploid 1C< 6pg	Diploid 1C≥ 6pg	Polyploid 1C< 6pg	Polyploid 1C≥ 6pg	Diploid 1C< 6pg	Diploid 1C≥ 6pg	Polyploid 1C< 6pg	Polyploid 1C≥ 6pg
Control	2.64 ±1.57	0.16 ±0.17	4.069 ±0.85	3.185 ±0.67	0.08 ±0.04	0.005 ±0	0.13 ±0.03	0.103 ±0.03
N	3.344 ±1.59	0.144 ±0.18	5.836 ±2.39	3.138 ±1.23	0.098 ±0.05	0.004 ±0.01	0.165 ±0.05	0.092 ±0.04
P	5.82 ±3.37	0.091 ±0.1	4.361 ±2.45	6.294 ±3.9	0.125 ±0.04	0.002 ±0	0.102 ±0.05	0.132 ±0.05
N+P	5.918 ±3.04	0.034 ±0.06	4.134 ±1.83	19.548 ±9.1	0.097 ±0.04	0.001 ±0	0.072 ±0.03	0.32 ±0.1

**(d)**	**S-weighted mean total biomass (g)**				**S-weighted mean biomass ratio**			
Treatment	Diploid 1C< 6pg	Diploid 1C≥ 6pg	Polyploid 1C< 6pg	Polyploid 1C≥ 6pg	Diploid 1C< 6pg	Diploid 1C≥ 6pg	Polyploid 1C< 6pg	Polyploid 1C≥ 6pg
Control	3.408 ±1.78	0.641 ±0.68	4.312 ±0.86	3.268 ±0.68	0.104 ±0.05	0.018 ±0.02	0.137 ±0.03	0.106 ±0.03
N	3.077 ±1.62	0.579 ±0.72	4.574 ±1.86	2.793 ±1.29	0.091 ±0.05	0.018 ±0.02	0.131 ±0.04	0.082 ±0.04
P	5.43 ±2.12	0.365 ±0.39	3.801 ±2.23	4.291 ±1.27	0.121 ±0.03	0.009 ±0.01	0.092 ±0.05	0.097 ±0.02
N + P	3.718 ±1.72	0.136 ±0.23	2.416 ±1.41	7.342 ±1.59	0.065 ±0.03	0.003 ±0	0.046 ±0.03	0.131 ±0.04

**(e)**	**R-weighted mean total biomass (g)**				**R-weighted mean biomass ratio**			
Treatment	Diploid 1C< 6pg	Diploid 1C≥ 6pg	Polyploid 1C< 6pg	Polyploid 1C≥ 6pg	Diploid 1C< 6pg	Diploid 1C≥ 6pg	Polyploid 1C< 6pg	Polyploid 1C≥ 6pg
Control	2.914 ±1.65	0.16 ±0.17	3.68 ±0.86	3.268 ±0.68	0.089 ±0.04	0.005 ±0	0.118 ±0.03	0.106 ±0.03
N	3.507 ±1.6	0.146 ±0.18	4.518 ±1.83	2.812 ±1.31	0.103 ±0.05	0.004 ±0.01	0.129 ±0.04	0.083 ±0.04
P	5.986 ±3.16	0.092 ±0.1	3.638 ±2.27	4.316 ±1.3	0.13 ±0.04	0.002 ±0	0.087 ±0.05	0.098 ±0.02
N+P	5.208 ±2.67	0.034 ±0.06	2.412 ±1.41	7.464 ±1.65	0.087 ±0.04	0.001 ±0	0.046 ±0.03	0.133 ±0.04

Table S12 Large GS ≥ 6 pg. Treatment contrasts and ANOVA output testing the effects and interactions of N, P, GS (small vs large, where large GS ≥ 6 pg), ploidy (diploid vs polyploid), on subplot: (a) total biomass; (b) total species numbers; (d) C-weighted biomass; (e) S-weighted biomass; and (f) R-weighted biomass testing for significance in treatment, GS, and ploidy. Dependent variables were square-root transformed. Part (c) shows multivariate ANOVA output where each C-S-R-weighted biomass are the response variables (i.e. n = 3). Baseline levels in the contrasts are: without N, without P, diploid, and small GS for N, P, GS and ploidy effects respectively (e.g. there is a 0.2 g increase in biomass with the addition of N, relative to subplots without N).

(a) Biomass

	Estimate	Std. error	t-value	Pr(>|t|)	ANOVA	df	F-value	*P*-value
(Intercept)	2.866	0.209	13.711	<0.0001	(Intercept)	1, 180	4150.617	<0.0001
N	0.173	0.302	0.571	0.5699	N	1, 60	6.504	0.0133
P	1.181	0.277	4.270	0.0001	P	1, 60	25.505	<0.0001
GS	-2.020	0.296	-6.833	<0.0001	GS	1, 180	115.179	<0.0001
ploidy	0.591	0.296	1.998	0.0472	ploidy	1, 180	365.676	<0.0001
N : P	-0.480	0.381	-1.259	0.2128	N : P	1, 60	0.996	0.3222
N : GS	-0.265	0.427	-0.619	0.5366	N : GS	1, 180	12.943	0.0004
P : GS	-1.379	0.391	-3.525	0.0005	P : GS	1, 180	8.740	0.0035
N : ploidy	0.167	0.427	0.390	0.6966	N : ploidy	1, 180	17.981	<0.0001
P : ploidy	-1.334	0.391	-3.410	0.0008	P : ploidy	1, 180	3.674	0.0569
GS : ploidy	1.665	0.418	3.982	0.0001	GS : ploidy	1, 180	484.650	<0.0001
N : P : GS	0.225	0.539	0.418	0.6766	N : P : GS	1, 180	17.100	0.0001
N : P : ploidy	-0.236	0.539	-0.437	0.6625	N : P : ploidy	1, 180	8.563	0.0039
N : GS : ploidy	-0.280	0.605	-0.463	0.6441	N : GS : ploidy	1, 180	26.225	<0.0001
P : GS : ploidy	2.221	0.553	4.017	0.0001	P : GS : ploidy	1, 180	91.794	<0.0001
N : P : GS : ploidy	2.700	0.762	3.544	0.0005	N : P : GS : ploidy	1, 180	12.563	0.0005

(b) Species number

	Estimate	Std. error	t-value	Pr(>|t|)	ANOVA	df	F-value	*P*-value
(Intercept)	4.470	0.097	46.096	<0.0001	(Intercept)	1, 180	6640.214	<0.0001
N	-0.522	0.140	-3.724	0.0004	N	1, 60	71.515	<0.0001
P	-0.534	0.128	-4.162	0.0001	P	1, 60	36.793	<0.0001
GS	-3.470	0.111	-31.120	<0.0001	GS	1, 180	1944.982	<0.0001
ploidy	-1.123	0.111	-10.077	<0.0001	ploidy	1, 180	46.763	<0.0001
N : P	-0.320	0.177	-1.812	0.0751	N : P	1, 60	5.919	0.018
N : GS	0.597	0.161	3.706	0.0003	N : GS	1, 180	71.875	<0.0001
P : GS	0.560	0.147	3.795	0.0002	P : GS	1, 180	64.411	<0.0001
N : ploidy	0.051	0.161	0.319	0.7498	N : ploidy	1, 180	0.211	0.6468

P : ploidy	0.103	0.147	0.696	0.4874
GS : ploidy	2.670	0.158	16.932	<0.0001
N : P : GS	-0.045	0.203	-0.219	0.8267
N : P : ploidy	-0.063	0.203	-0.310	0.7572
N : GS : ploidy	-0.286	0.228	-1.256	0.2107
P : GS : ploidy	-0.066	0.209	-0.315	0.7529
N : P : GS : ploidy	0.274	0.287	0.954	0.3411

P : ploidy	1, 180	2.303	0.1308
GS : ploidy	1, 180	1420.937	<0.0001
N : P : GS	1, 180	0.415	0.5201
N : P : ploidy	1, 180	0.267	0.6061
N : GS : ploidy	1, 180	0.571	0.4507
P : GS : ploidy	1, 180	0.301	0.5837
N : P : GS : ploidy	1, 180	0.911	0.3411

(c) C-S-R

	Estimate	Std. error	t-value	Pr(>|t|)
(Intercept)	4.953	0.352	14.090	<0.0001
N	0.302	0.508	0.594	0.5545
P	2.051	0.465	4.411	<0.0001
GS	-3.571	0.497	-7.183	<0.0001
ploidy	1.028	0.497	2.067	0.0402
N : P	-0.879	0.641	-1.372	0.1751
N : GS	-0.452	0.719	-0.629	0.5303
P : GS	-2.374	0.658	-3.610	0.0004
N : ploidy	0.278	0.719	0.387	0.6993
P : ploidy	-2.323	0.658	-3.532	0.0005
GS : ploidy	2.962	0.703	4.214	<0.0001
N : P : GS	0.462	0.906	0.511	0.6103
N : P : ploidy	-0.419	0.906	-0.463	0.6441
N : GS : ploidy	-0.488	1.017	-0.480	0.6319
P : GS : ploidy	3.799	0.930	4.085	0.0001
N : P : GS : ploidy	4.379	1.281	3.418	0.0008

ANOVA	df	F-value	*P*-value
(Intercept)	1, 180	4290.323	<0.0001
N	1, 60	4.866	0.0312
P	1, 60	23.654	<0.0001
GS	1, 180	132.048	<0.0001
ploidy	1, 180	375.223	<0.0001
N : P	1, 60	0.549	0.4614
N : GS	1, 180	12.562	0.0005
P : GS	1, 180	8.331	0.0044
N : ploidy	1, 180	15.594	0.0001
P : ploidy	1, 180	2.540	0.1127
GS : ploidy	1, 180	503.693	<0.0001
N : P : GS	1, 180	17.141	0.0001
N : P : ploidy	1, 180	7.638	0.0063
N : GS : ploidy	1, 180	24.229	<0.0001
P : GS : ploidy	1, 180	91.136	<0.0001
N : P : GS : ploidy	1, 180	11.684	0.0008

(d) C

	Estimate	Std. error	t-value	Pr(>|t|)
(Intercept)	1.543	0.158	9.780	<0.0001
N	0.217	0.228	0.950	0.346
P	0.789	0.209	3.780	0.0004
GS	-1.197	0.214	-5.596	<0.0001
ploidy	0.464	0.214	2.169	0.0314
N : P	-0.207	0.287	-0.719	0.4748
N : GS	-0.255	0.309	-0.824	0.411
P : GS	-0.871	0.283	-3.076	0.0024
N : ploidy	0.149	0.309	0.482	0.6302
P : ploidy	-0.778	0.283	-2.748	0.0066

ANOVA	df	F-value	*P*-value
(Intercept)	1, 180	2303.550	<0.0001
N	1, 60	24.675	<0.0001
P	1, 60	39.104	<0.0001
GS	1, 180	59.277	<0.0001
ploidy	1, 180	412.279	<0.0001
N : P	1, 60	3.626	0.0617
N : GS	1, 180	14.184	0.0002
P : GS	1, 180	14.040	0.0002
N : ploidy	1, 180	28.980	<0.0001
P : ploidy	1, 180	10.942	0.0011

GS : ploidy	0.965	0.303	3.190	0.0017	GS : ploidy	1, 180	426.734	<0.0001
N : P : GS	0.105	0.390	0.268	0.7889	N : P : GS	1, 180	19.245	<0.0001
N : P : ploidy	-0.186	0.390	-0.478	0.6332	N : P : ploidy	1, 180	11.100	0.001
N : GS : ploidy	-0.145	0.438	-0.332	0.7402	N : GS : ploidy	1, 180	35.499	<0.0001
P : GS : ploidy	1.499	0.400	3.743	0.0002	P : GS : ploidy	1, 180	93.559	<0.0001
N : P : GS : ploidy	2.210	0.551	4.008	0.0001	N : P : GS : ploidy	1, 180	16.061	0.0001

(e) S

	Estimate	Std. error	t-value	Pr(>|t|)	ANOVA	df	F-value	*P*-value
(Intercept)	1.779	0.113	15.761	<0.0001	(Intercept)	1, 180	4258.283	<0.0001
N	-0.093	0.163	-0.568	0.5722	N	1, 60	2.876	0.0951
P	0.513	0.149	3.433	0.0011	P	1, 60	2.783	0.1005
GS	-1.088	0.160	-6.818	<0.0001	GS	1, 180	123.331	<0.0001
ploidy	0.287	0.160	1.799	0.0737	ploidy	1, 180	264.659	<0.0001
N : P	-0.319	0.206	-1.551	0.1261	N : P	1, 60	0.089	0.7663
N : GS	0.017	0.231	0.075	0.9405	N : GS	1, 180	12.874	0.0004
P : GS	-0.675	0.211	-3.195	0.0017	P : GS	1, 180	5.309	0.0224
N : ploidy	0.127	0.231	0.550	0.5833	N : ploidy	1, 180	10.769	0.0012
P : ploidy	-0.711	0.211	-3.365	0.0009	P : ploidy	1, 180	0.910	0.3414
GS : ploidy	0.820	0.226	3.633	0.0004	GS : ploidy	1, 180	357.551	<0.0001
N : P : GS	0.112	0.291	0.385	0.7006	N : P : GS	1, 180	10.285	0.0016
N : P : ploidy	-0.083	0.291	-0.285	0.776	N : P : ploidy	1, 180	5.104	0.0251
N : GS : ploidy	-0.216	0.326	-0.663	0.5083	N : GS : ploidy	1, 180	12.012	0.0007
P : GS : ploidy	1.127	0.299	3.775	0.0002	P : GS : ploidy	1, 180	68.859	<0.0001
N : P : GS : ploidy	1.095	0.411	2.662	0.0085	N : P : GS : ploidy	1, 180	7.088	0.0085

(f) R

	Estimate	Std. error	t-value	Pr(>|t|)	ANOVA	df	F-value	*P*-value
(Intercept)	1.630	0.115	14.152	<0.0001	(Intercept)	1, 180	3932.601	<0.0001
N	0.178	0.167	1.069	0.2892	N	1, 60	0.668	0.4171
P	0.749	0.152	4.917	<0.0001	P	1, 60	13.456	0.0005
GS	-1.285	0.163	-7.886	<0.0001	GS	1, 180	197.863	<0.0001
ploidy	0.276	0.163	1.695	0.0918	ploidy	1, 180	272.001	<0.0001
N : P	-0.353	0.210	-1.682	0.0978	N : P	1, 60	0.122	0.7279
N : GS	-0.214	0.236	-0.909	0.3645	N : GS	1, 180	5.530	0.0198
P : GS	-0.828	0.216	-3.842	0.0002	P : GS	1, 180	2.649	0.1054
N : ploidy	0.002	0.236	0.009	0.9931	N : ploidy	1, 180	3.106	0.0797
P : ploidy	-0.834	0.216	-3.869	0.0002	P : ploidy	1, 180	0.173	0.6777
GS : ploidy	1.177	0.230	5.107	<0.0001	GS : ploidy	1, 180	520.549	<0.0001

N : P : GS	0.246	0.297	0.828	0.4087	N : P : GS	1, 180	13.896	0.0003
N : P : ploidy	-0.150	0.297	-0.505	0.6144	N : P : ploidy	1, 180	3.397	0.067
N : GS : ploidy	-0.126	0.333	-0.379	0.7055	N : GS : ploidy	1, 180	14.417	0.0002
P : GS : ploidy	1.173	0.305	3.847	0.0002	P : GS : ploidy	1, 180	68.758	<0.0001
N : P : GS : ploidy	1.074	0.420	2.557	0.0114	N : P : GS : ploidy	1, 180	6.537	0.0114

Methods S1 Phylogenetic tree of species present at Park Grass, used to estimate the correlation structure in phylogenetic generalised mixed model and *pgls* analyses, in Newick format.

(((((((((((((PT:1.0,PP:1.0):1.0,HP:2.0):1.0,AP:3.0):1.0,DC:4.0):4.0,(CR:3.0,(FR:2.0,(LM:1.0,FP:1.0):1.0):1.0):5.0):1.0,DG:9.0):1.0,HL:10.0):5.0,((BM:1.0,AO:1.0):3.0,(AC:2.0,(TF:1.0,AE:1.0):1.0):2.0):11.0):1.0,BH:16.0):3.0,(LC:2.0,(CY:1.0,CX:1.0):1.0):17.0):1.0,FM:20.0):39.0,((((RB:1.0,RA:1.0):1.0,RU:2.0):1.0,RF:3.0):35.0,(((((((TR:1.0,TP:1.0):1.0,OR:2.0):1.0,LP:3.0):1.0,LO:4.0):3.0,(AU:2.0,(SM:1.0,PX:1.0):1.0):5.0):2.0,(VR:1.0,QR:1.0):8.0):25.0,(((SG:1.0,CF:1.0):1.0,RC:2.0):22.0,(PV:21.0,(((((SO:1.0,PZ:1.0):1.0,AR:2.0):2.0,(VC:1.0,PL:1.0):3.0):1.0,GV:5.0):15.0,(((((PS:1.0,HS:1.0):1.0,CM:2.0):1.0,AS:3.0):1.0,KA:4.0):10.0,(BP:9.0,(AM:8.0,(CN:7.0,(TG:6.0,(HI:5.0,((TO:1.0,CC:1.0):3.0,(LH:2.0,(LA:1.0,HR:1.0):1.0):2.0):1.0):1.0):1.0):1.0):1.0):5.0):6.0):1.0):3.0):10.0):4.0):21.0):1.0,OV:60.0);

References

Albach DC, Greilhuber J. 2004. Genome size variation and evolution in *Veronica*. *Annals of Botany* **94:** 897–911.

Barow M, Meister A. 2003. Endopolyploidy in seed plants is differently correlated to systematics, organ, life strategy and genome size. *Plant, Cell & Environment* **26:** 571–584.

Bennett MD, Leitch IJ. 2012. *Plant DNA C-values database (release 6.0, Dec. 2012)*. URL http://data.kew.org/cvalues/

Bennett MD, Smith JB, Lewis Smith RI. 1982. DNA amounts of angiosperms from the Antarctic and South Georgia. *Environmental and Experimental Botany* **22:** 307–318.

Cerbah M, Coulaud J, Brown SC, Siljak-Yakovlev S. 1999. Evolutionary DNA variation in the genus *Hypochaeris*. *Heredity* **82:** 261–266.

Crawley MJ, Johnston AE, Silvertown J, Dodd M, de Mazancourt C, Heard MS, Henman DF, Edwards GR. 2005. Determinants of species richness in the Park Grass Experiment. *The American Naturalist* **165:** 179–192.

Evans GM, Rees H, Snell CL, Sun S. 1972. The relationship between nuclear DNA amount and the duration of the mitotic cycle. *Chromosomes Today* **3:** 24–31.

Favre JM, Brown S. 1996. A flow cytometric evaluation of the nuclear DNA content and GC percent in genomes of European oak species. *Annales des Sciences Forestieres* **53:** 915–917.

Grime JP, Shacklock JML, Band SR. 1985. Nuclear DNA contents, shoot phenology and species co-existence in a limestone grassland community. *New Phytologist* **100:** 435–445.

Leitch IJ, Beaulieu JM, Cheung K, Hanson L, Lysak M, Fay MF. 2007. Punctuated genome size evolution in Liliaceae. *Journal of Evolutionary Biology* **20:** 2296–2308.

Mowforth MA.1986. *Variation in nuclear DNA amounts in flowering plants: an ecological analysis*. PhD thesis, University of Sheffield, Sheffield, UK.

Murray BG, De Lange PJ, Ferguson AR. 2005. Nuclear DNA variation, chromosome numbers and polyploidy in the endemic and indigenous grass flora of New Zealand. *Annals of Botany* **96:** 1293–1305.

Olszewska MJ, Osiecka R. 1982. The relationship between 2C DNA content, life-cycle type, systematic position, and the level of DNA endoreplication in nuclei of parenchyma cells during growth and differentiation of roots in some monocotyledonous species. *Biochemie Und Physiologie Der Pflanzen* **177:** 319–336.

Olszewska MJ, Osiecka R. 1983. The relationship between 2C DNA content, life-cycle type, systematic position and the dynamics of DNA endoreplication in parenchyma nuclei during growth and differentiation of roots in some dicotyledonous-herbaceous species. *Biochemie Und Physiologie Der Pflanzen* **178:** 581–599.

Paradis E, Claude J, Strimmer K. 2004. APE: analyses of phylogenetics and evolution in R language. *Bioinformatics* **20:** 289–290.

Pinheiro J, Bates D, DebRoy S, Sarkar D, R Core Team. 2013. *nlme: Linear and Nonlinear Mixed Effects Models*. R package version 3.1-104. URL http://cran.r-project.org/package=nlme

Pires JC, Lim KY, Kovarik A, Matyasek R, Boyd A, Leitch AR, Leitch IJ, Bennett MD, Soltis PS, Soltis DE. 2004. Molecular cytogenetic analysis of recently evolved *Tragopogon* (Asteraceae) allopolyploids reveal a karyotype that is additive of the diploid progenitors. *American Journal of Botany* **91:** 1022–1035.

Roser M. 1995. Typification of the names of two widespread Eurasian grass species: *Avena pratensis* L. and *A. pubescens* Huds. (Poaceae: Helitotrichon) *Taxon* **44:** 395–399.

Siljak-Yakovlev S, Pustahija F, Šolic EM, Bogunic F, Muratovic E, Bašic N, Catrice O, Brown SC. 2010. Towards a genome size and chromosome number database of Balkan flora: C-values in 343 taxa with novel values for 242. *Advanced Science Letters* **3:** 190–213.

Šmarda P, Bureš P, Horová L, Foggi B, Rossi G. 2008. Genome size and GC content evolution of *Festuca*: ancestral expansion and subsequent reduction. *Annals of Botany* **101:** 421–433.

Šmarda P, Hejcman M, Březinová A, Horová L, Steigerová H, Zedek F, Bureš P, Hejcmanová P, Schellberg J. 2013. Effect of phosphorus availability on the selection of species with different ploidy levels and genome sizes in a long-term grassland fertilization experiment. *New Phytologist* **200:** 911–921.

Smith JB, Bennett MD. 1975. DNA variation in the genus *Ranunculus*. *Heredity* **35:** 231–239.

Temsch EM, Greilhuber J. 2010. Genome size in Dipsacaceae and *Morina longifolia* (Morinaceae). *Plant Systematics and Evolution* **289:** 45–56.

Temsch EM, Temsch W, Ehrendorfer-Schratt L, Greilhuber J. 2010. Heavy metal pollution, selection, and genome size: the species of the Žerjav study revisited with flow cytometry. *Journal of Botany* **2010:** article ID 596542.
